# Supplementary material for: Paralog dispensability shapes homozygous deletion patterns in tumor genomes
Source: Mol Syst Biol. 2023 Nov 14;19(12):e11987. doi: 10.15252/msb.202311987 (PMC10698506; doi:10.15252/msb.202311987)
Supplement: Supplementary file 1 — Expanded View Figures PDF [file MSB-19-e11987-s005.pdf]

Expanded View Figures

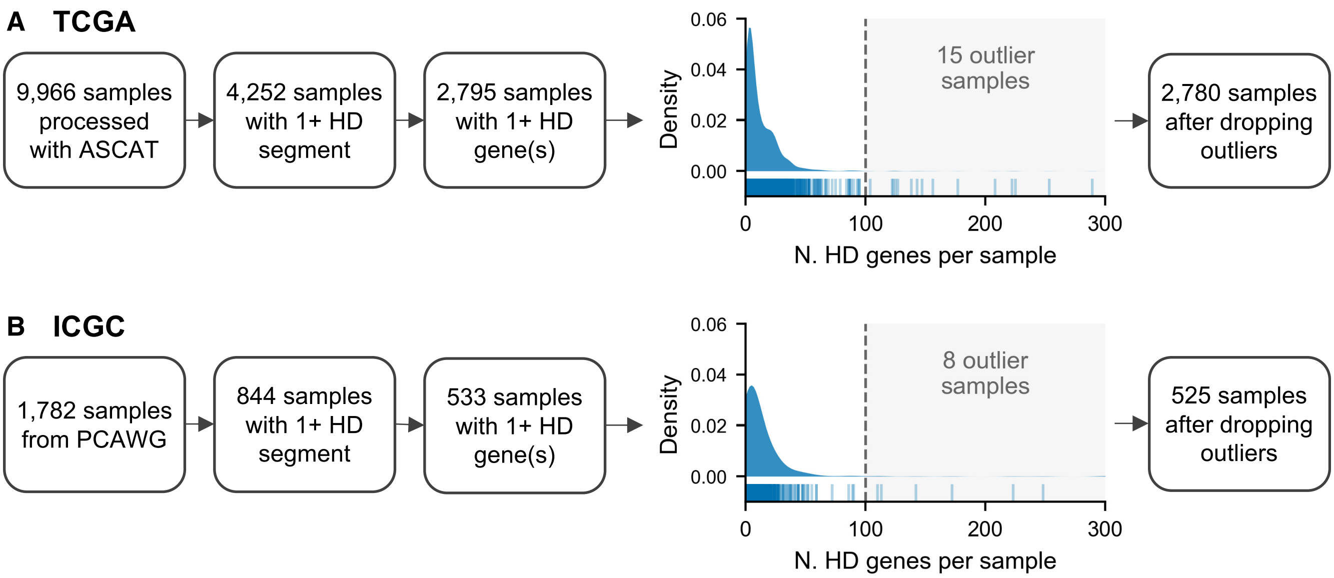

Figure EV1. Workflow for tumor samples used in this study.

A Flowchart for the number of TCGA tumor samples used for HD analysis. The density/tick plot shows the distribution of the number of HD genes per sample; only samples with at least 1 gene HD are shown in this plot. Samples to the right of the dotted line were marked as outliers and dropped from further analysis.

B Same as (A) but for ICGC tumor samples from the PCAWG study.

Figure EV2. Gene-level homozygous deletion frequency across the genome.

Line plots showing, for each gene plotted according to its genomic location, the number of TCGA tumor samples in which the gene is fully homozygously deleted. The number of samples is capped at 40 for visualization purposes. Orange ticks show the location of all TSGs with at least three HDs; four TSG peaks are annotated with the gene symbol and number of HDs. Fragile sites are denoted by blue ticks and centromeres by dotted gray lines.

© 2023 The Authors

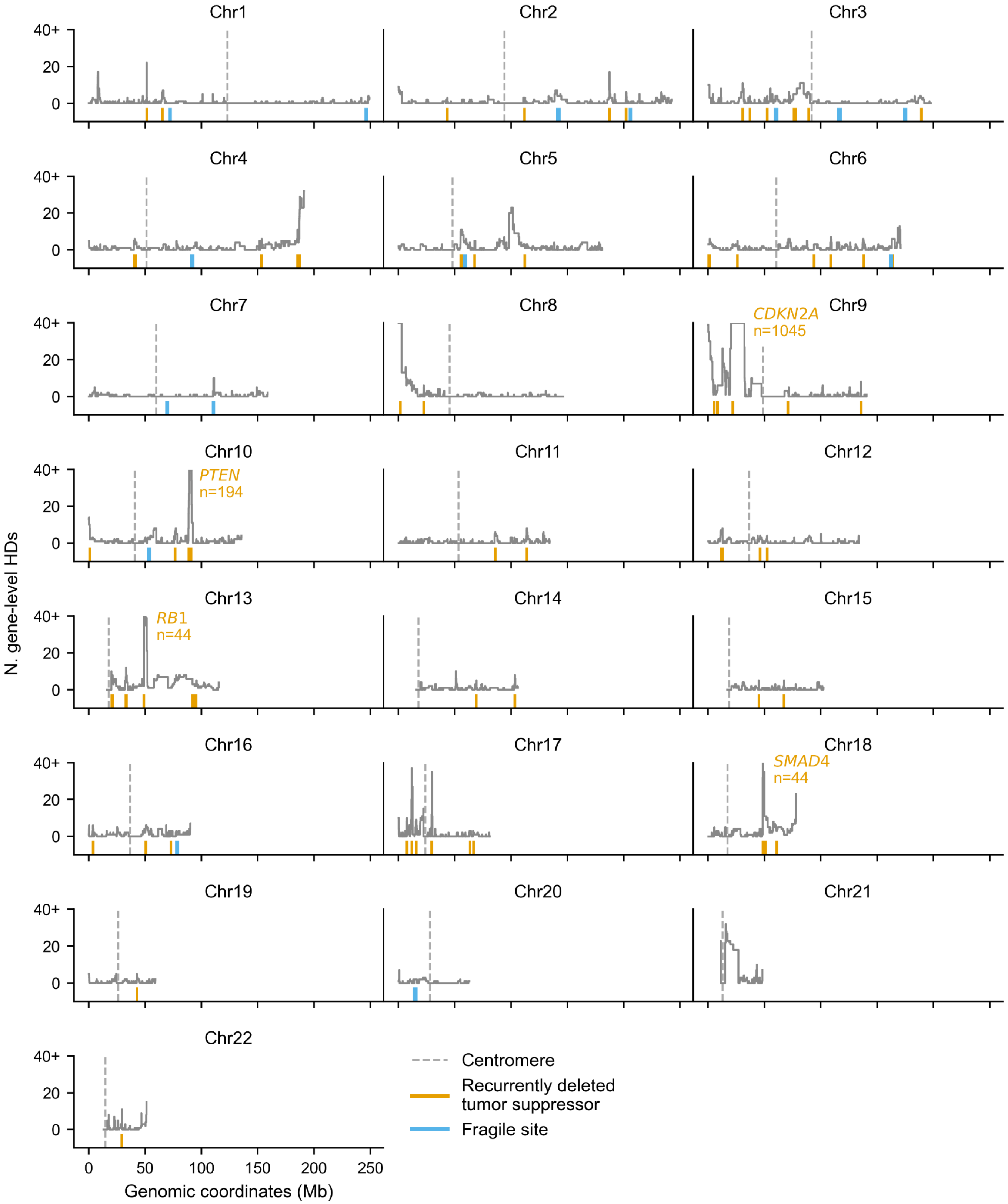
Chr1
Chr2
Chr3
Chr4
Chr5
Chr6
Chr7
Chr8
Chr9
CDKN2A
n=1045
Chr10
PTEN
n=194
Chr11
Chr12
Chr13
RB1
n=44
Chr14
Chr15
Chr16
Chr17
Chr18
SMAD4
n=44
Chr19
Chr20
Chr21
Chr22
40+
20
0
N. gene-level HDs
0
50
100
150
200
250
Genomic coordinates (Mb)
Centromere
Recurrently deleted tumor suppressor
Fragile site

Figure EV2.

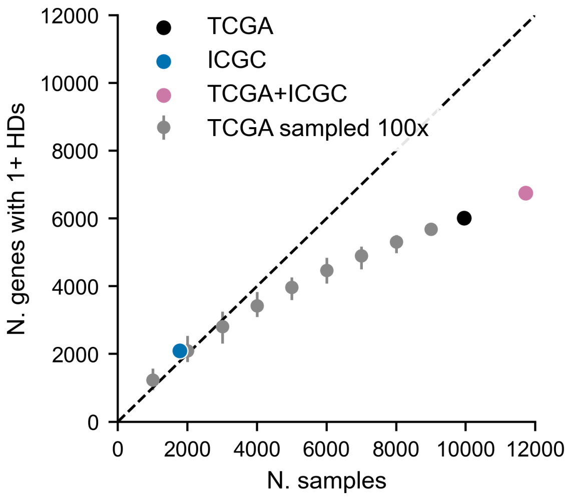

Figure EV3. Gene HD saturation analysis.

Dot plot showing the number of unique gene HDs observed (y-axis) for increasing numbers of tumor samples (x-axis). The black dot shows the actual number of unique gene HDs observed in the TCGA cohort. Gray dots are the result of down-sampling the TCGA cohort and error bars indicate the minimum and maximum values observed from 100 random samplings. The blue dot shows the actual number of unique gene HDs observed in the ICGC cohort, while the pink dot indicates the number of gene HDs that are observed when combining the TCGA and ICGC cohorts into one dataset.

Figure EV4. Paralog passengers are more likely to be subject to homozygous but not hemizygous deletion.

A Similar to Fig 2A but for TCGA tumor samples stratified by cancer type. Top: Percentage of passenger (non-driver) genes that are deleted in either zero (0) or at least one (1+) TCGA samples. The number of TCGA tumor samples for each cancer type considered is shown. Bottom: For passenger genes grouped according to never deleted (0) or deleted at least once (1+), the solid blue line shows the percentage of genes in each group that are paralogs. The dashed line shows the percentage of all passenger genes that are paralogs. Annotations show the Odds Ratio (OR) for a Fisher's Exact Test comparing the percentage of paralogs among genes with 0 vs. 1+ HDs; asterisk (*) indicates $P < 0.05$.

B Boxplots showing the number of TCGA samples in which gene-level LOH is observed for singleton vs. paralog passenger genes. LOH here is identified when one allele has copy number equal to 0. From left to right the box plots show all LOH segments, only focal LOH segments, copy loss segments (total copy number = 1), and copy neutral LOH segments (total copy number = 2). The boxes represent the first and third quartiles (Q1 and Q3) of the distribution, the horizontal black line the median, and the whiskers extend up to 1.5*the interquartile range past Q1 and Q3. Outliers are shown as gray circles. The P-values shown are from MWU tests comparing paralogs and singletons.

C Same as (B) but for ICGC tumor samples.

© 2023 The Authors

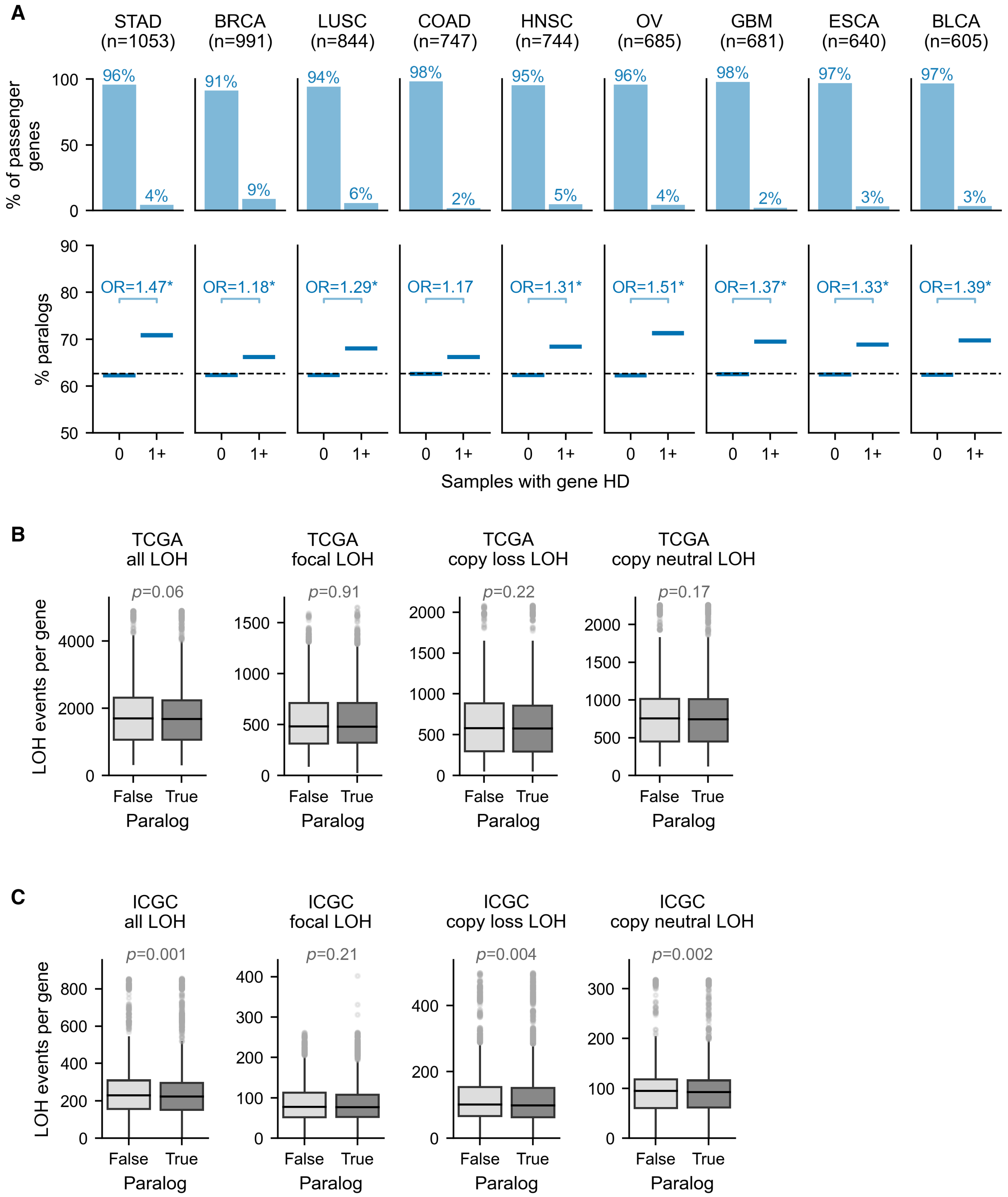
A
STAD (n=1053)
BRCA (n=991)
LUSC (n=844)
COAD (n=747)
HNSC (n=744)
OV (n=685)
GBM (n=681)
ESCA (n=640)
BLCA (n=605)
% of passenger genes
96% 4%
91% 9%
94% 6%
98% 2%
95% 5%
96% 4%
98% 2%
97% 3%
97% 3%
% paralogs
OR=1.47*
OR=1.18*
OR=1.29*
OR=1.17
OR=1.31*
OR=1.51*
OR=1.37*
OR=1.33*
OR=1.39*
Samples with gene HD
B
TCGA all LOH
TCGA focal LOH
TCGA copy loss LOH
TCGA copy neutral LOH
p=0.06
p=0.91
p=0.22
p=0.17
LOH events per gene
False True
Paralog
C
ICGC all LOH
ICGC focal LOH
ICGC copy loss LOH
ICGC copy neutral LOH
p=0.001
p=0.21
p=0.004
p=0.002

Figure EV4.

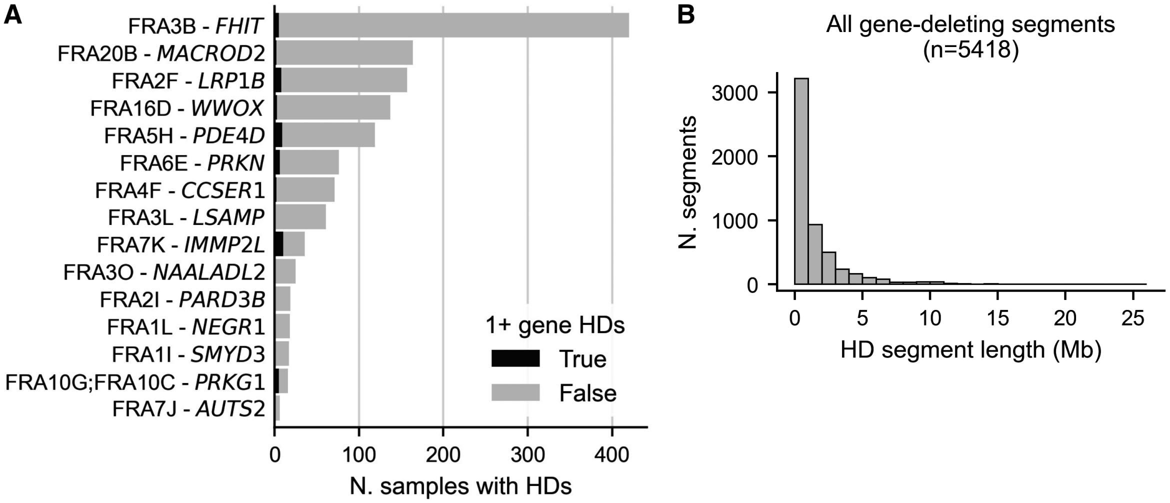

Figure EV5. Characterization of HD segments.

A Bar plot showing the number of TCGA samples with an HD overlapping each of 15 major fragile sites, with the section of the bar colored black indicating the number of HDs that result in at least 1 full gene HD. Fragile sites are listed with their name (e.g. FRA3B) and the longest gene they contain (e.g. *FHIT*).

B Histogram showing the distribution of the lengths of all HD segments from both cohorts that fully delete at least 1 gene.

 © 2023 The Authors
