# Supplementary material for: Paralog dispensability shapes homozygous deletion patterns in tumor genomes
Source: Mol Syst Biol. 2023 Nov 14;19(12):e11987. doi: 10.15252/msb.202311987 (PMC10698506; doi:10.15252/msb.202311987)
Supplement: Supplementary file 5 — PDF+ [file MSB-19-e11987-s006.pdf]

Article

TRANSPARENT PROCESS | OPEN ACCESS

molecular systems biology

Paralog dispensability shapes homozygous deletion patterns in tumor genomes

Barbara De Kegel[1] & Colm J Ryan[1,2,*]

Abstract

Genomic instability is a hallmark of cancer, resulting in tumor genomes having large numbers of genetic aberrations, including homozygous deletions of protein coding genes. That tumor cells remain viable in the presence of such gene loss suggests high robustness to genetic perturbation. In model organisms and cancer cell lines, paralogs have been shown to contribute substantially to genetic robustness—they are generally more dispensable for growth than singletons. Here, by analyzing copy number profiles of > 10,000 tumors, we test the hypothesis that the increased dispensability of paralogs shapes tumor genome evolution. We find that genes with paralogs are more likely to be homozygously deleted and that this cannot be explained by other factors known to influence copy number variation. Furthermore, features that influence paralog dispensability in cancer cell lines correlate with paralog deletion frequency in tumors. Finally, paralogs that are broadly essential in cancer cell lines are less frequently deleted in tumors than non-essential paralogs. Overall, our results suggest that homozygous deletions of paralogs are more frequently observed in tumor genomes because paralogs are more dispensable.

Keywords copy number variation; dispensability; paralogs; robustness; tumor genome evolution

Subject Categories Cancer; Chromatin, Transcription & Genomics; Computational Biology

DOI 10.15252/msb.202311987 | Received 5 September 2023 | Revised 24 October 2023 | Accepted 26 October 2023 | Published online 14 November 2023

Mol Syst Biol. (2023) 19: e11987

Introduction

Tumor genomes typically abound with genetic aberrations, ranging from missense mutations in individual genes to deletions of entire chromosome arms (Vogelstein *et al*, 2013). These genetic alterations can result in reduced functionality, or complete loss of function, of multiple proteins in any given tumor cell. Despite these alterations, tumor cells remain viable and even thrive, suggesting that they are highly robust to genetic perturbation. This raises an important question: How do tumor cells tolerate such gene loss? One potential explanation is paralog buffering.

Paralogs are genes that arose from gene duplication events, the primary means by which new genes are created (Zhang, 2003). In multiple model organisms, paralogs have been demonstrated to be more dispensable than singletons (genes without a paralog) (Gu *et al*, 2003; Kamath *et al*, 2003; White *et al*, 2013). There are a number of reasons for why a paralog might be more dispensable than a singleton gene, including preferential retention of duplications of non-essential genes (He & Zhang, 2006; O'Toole *et al*, 2018), but perhaps the most obvious explanation is buffering between paralogs. Many paralog pairs retain at least some degree of functional redundancy, even after long evolutionary periods, which may allow them to buffer each other's loss (Kuzmin *et al*, 2021). Consistent with this model, systematic double gene deletion studies in budding yeast have revealed that ~30% of paralog pairs display negative genetic interactions, where the combined gene deletion causes a greater than expected fitness defect, indicative of a buffering relationship (Dean *et al*, 2008; DeLuna *et al*, 2008; VanderSluis *et al*, 2010).

We, and others, have made similar observations using loss of function screens in human cancer cell lines—paralogs are generally more dispensable for cellular growth than singletons, and this is even more evident for highly sequence similar paralogs and genes with multiple paralogs (Wang *et al*, 2015; Dandage & Landry, 2019; De Kegel & Ryan, 2019; Dede *et al*, 2020). Furthermore, many members of paralog pairs can be lost individually but not in combination, suggesting that their dispensability can be directly attributed to paralog buffering (De Kegel & Ryan, 2019; Dede *et al*, 2020; De Kegel *et al*, 2021). While it is therefore clear that paralogs contribute to the genetic robustness of tumor cell lines *in vitro*, it is not clear whether this is a significant factor in the genetic robustness of tumors *in vivo*.

One means to explore the impact of paralogs on genetic robustness in tumors *in vivo* is through the analysis of compendia of tumor genomes, which provide a record of those genetic alterations that can be tolerated by tumor cells, under at least some circumstances. Tumors evolve through the accumulation of somatic alterations followed by clonal selection. Alterations that increase cellular fitness will likely be observed more frequently across tumor genomes, as positive selection would increase their prevalence in

1 School of Computer Science and Systems Biology Ireland, University College Dublin, Dublin, Ireland
2 Conway Institute of Biomolecular and Biomedical Research, University College Dublin, Dublin, Ireland
*Corresponding author. Tel: +353-1-716-7777; E-mail: colm.ryan@ucd.ie

© 2023 The Authors. Published under the terms of the CC BY 4.0 license.

any given tumor population. Conversely, deleterious alterations will likely be observed less frequently across tumor genomes, as negative selection would cause clonal lineages with such aberrations to die out. If paralog genes are more dispensable for tumor cells *in vivo*, we expect that in general deleterious alterations of paralog genes will be under weaker negative selection than similar alterations to singleton genes. As the majority (> 60%) of genes in the human genome have at least one paralog (Zerbino *et al*, 2018), paralog dispensability has the potential to substantially shape tumor genomes.

Analyses of selection in cancer have largely focused on positive selection—a key aim has been to distinguish driver alterations, that directly promote tumorigenesis, from passenger alterations, that provide no fitness benefit and are presumed to be present due to "hitchhiking" with driver alterations (Vogelstein *et al*, 2013; Lawrence *et al*, 2014). More recently, several dN/dS-based approaches—which compare the expected number of nonsynonymous mutations (dN) to the expected number of synonymous mutations (dS) within a gene—have been used to identify signals of negative selection. In general, evidence for selection against missense mutations has been challenging to detect. In diploid and tetraploid regions even nonsense mutations in broadly essential genes appear to be well-tolerated, but in haploid regions, clear patterns of selection against nonsense mutations have been detected (Van den Eynden *et al*, 2016; Martincorena *et al*, 2017; Weghorn & Sunyaev, 2017; López *et al*, 2020), suggesting that most non-driver genes are haplosufficient in cancer cells.

Here, we focus our analysis on homozygous gene deletions—genetic aberrations that are guaranteed to result in complete protein loss. Homozygous deletion (HD) frequency has been previously used to identify tumor suppressor genes, whose loss recurs across tumor genomes due to positive selection (Zack *et al*, 2013; Cheng *et al*, 2017); but here we are particularly interested in HD frequency of non-driver (i.e., passenger) genes, whose loss will likely not provide a selective advantage. In the absence of positive selection, recurrent HDs may still be observed due to localized decreased negative selection strength and/or increased HD generation rate, which can occur at fragile sites or near telomeres (Bignell *et al*, 2010; Cheng *et al*, 2017). If paralog HDs are subject to weaker negative selection, this should be observable as a higher frequency of HDs among paralog compared with singleton genes—assuming equal rates of HD generation. In other words, we expect that tumor clones with stochastically acquired HDs of singleton genes will expire at a higher rate than clones that acquired HDs of paralog genes, leading to different observed frequencies of these types of HDs across tumor genomes (Fig 1).

To investigate patterns of HDs, we perform a systematic analysis of homozygous deletions in 9,951 tumor samples from The Cancer Genome Atlas (Cancer Genome Atlas Research Network *et al*, 2013) and 1,774 tumor samples from the International Cancer Genome Consortium (ICGC/TCGA Pan-Cancer Analysis of Whole Genomes Consortium, 2020). We show that among non-driver genes, HDs are more likely to be observed in paralogs than singletons and that this is not solely due to factors known to influence HD generation, such as proximity to recurrently deleted tumor suppressors or fragile sites. Furthermore, we find that properties of paralogs previously shown to influence their dispensability in cancer cell lines also influence their homozygous deletion frequency in tumors, for example, HDs are more likely to be observed in genes that have a larger number of paralogs. Finally, we show that HDs are less likely to be observed for essential paralogs than non-essential paralogs, suggesting that the increased HD frequency of paralogs is due to their generally higher dispensability rather than being a general feature of paralogs.

In addition to supporting the hypothesis that paralogs are a significant source of robustness in tumors, our results provide insight into the homozygous deletion patterns observed in tumors and highlight constraints operating on gene loss in tumor cells.

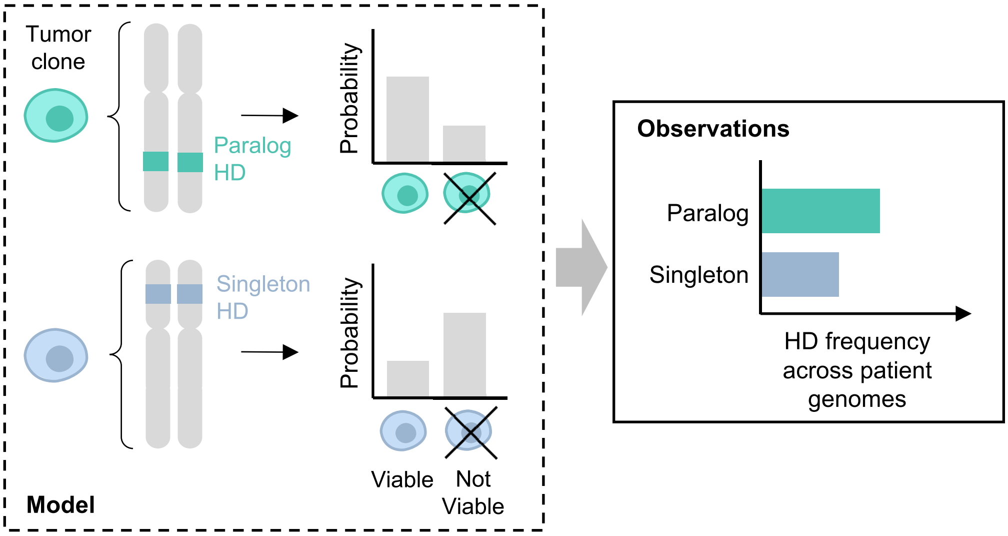

Figure 1. Theoretical model for the observed HD frequencies of paralog and singleton passenger genes.
Tumor clones with homozygous deletion of a paralog gene are more likely to be viable than tumor clones with homozygous deletion of a singleton gene. This leads to a comparatively higher frequency of paralog HDs across patient samples.

 © 2023 The Authors

Results

Homozygous deletions are more likely to be observed for paralog than singleton passenger genes

To assess the homozygous deletion frequency of paralog and singleton genes, we obtained allele-specific copy number profiles for 9,966 tumor samples spanning 33 cancer types from the Cancer Genome Atlas (TCGA) (see Materials and Methods). Among these samples, less than half (4,252) had at least one autosomal homozygous deletion (HD) segment—defined as a segment of the genome where the copy number of both alleles is zero—and fewer again had a HD of a protein-coding gene. To ensure that only complete protein loss was considered, we conservatively called protein-coding genes as deleted when the full coding sequence of their longest associated transcript was deleted. After removing a small number of "hyper-deleted" samples (see Materials and Methods), we retained 9,951 samples for further analysis. Among these found that 2,780 samples contained an HD of at least one autosomal protein coding gene (Fig EV1A and Dataset EV1). Typically, an HD that results in the loss of a protein coding gene also results in the loss of several chromosomally adjacent genes—in this dataset a median of three genes are lost per gene-deleting HD segment.

We visualized gene HD frequency across the whole genome and observed that, consistent with expectation, several well-known tumor suppressor genes (TSGs), including *CDKN2A*, *PTEN*, *RB1*, and *SMAD4* are associated with peaks of recurrent HDs (Fig EV2). To identify these genes systematically, we assembled a list of 652 known cancer driver genes (TSGs and oncogenes) by combining the Cancer Gene Census (Sondka *et al*, 2018) with a comprehensive set of driver genes identified in the TCGA (Bailey *et al*, 2018) (see Materials and Methods). We found that almost half (49%) of the HDs that result in the loss of at least one protein coding gene overlap a known tumor suppressor. A total of 188 (46%) of the 411 annotated TSGs are fully homozygously deleted at least once in the TCGA samples, while 77 are recurrently homozygously deleted (in three or more samples). As our primary interest is not in driver genes, and as they are likely subject to different selection pressures than other genes, we excluded them from our analysis. The exclusion of all 652 driver genes, plus 16 genes whose coding regions overlap the coding regions of these driver genes, left us with 16,898 genes, that we refer to as passengers, for further analysis (see Materials and Methods).

We find that most of these genes are never homozygously deleted (Fig 2A, top). Specifically, ~66% of all passenger genes have no HDs in any TCGA tumor samples, while only ~9% of passenger genes are recurrently deleted—defined as having HDs in at least three samples. We note that due to the limited sample size, this is likely an underestimate of the number of passenger genes that can be deleted in tumors. To estimate the level of saturation we sub-sample the TCGA dataset and plot the number of unique genes with at least one observed HD for increasing sample sizes (Fig EV3). We observe that up to ~3,000 samples the number of unique gene HDs increases linearly with sample size and thereafter the rate of newly seen gene HDs starts to decrease, with one new gene HD being observed for every 2–3 samples added to the cohort. However, we are not close to saturation, meaning that it is highly likely that some passenger genes are never homozygously deleted due to chance rather than negative selection.

Genome-wide, it is clear that gene HD frequency is generally low, with a small number of highly deleted regions corresponding to TSG locations (Fig EV2). This paucity of HDs is in line with a previous analysis of a different set of tumor samples which showed that compared to expectation (based on hemizygous deletion frequency), HDs are generally strongly depleted across the genome, likely due to negative selection (Cheng *et al*, 2017). As the frequency of passenger gene HD is low (median zero HDs per passenger gene) and highly skewed, we focus our analysis on the odds of observing any HD and recurrent HD.

Grouping passenger genes according to the number of samples in which they are homozygously deleted, we find that, compared to singletons, paralogs are significantly enriched among passenger genes that are deleted at least once (Fisher's Exact Test (FET): Odds Ratio = 1.30, P = 4e-15) and further enriched among recurrently deleted genes (FET: OR = 1.43, P = 2e-9; Fig 2A). We validate this finding in an independent cohort of 1,774 tumor samples from the International Cancer Genome Consortium (ICGC) (Fig 2B and Dataset EV2; see Materials and Methods). As this cohort is smaller, fewer passenger genes (~12%) are observed to be homozygously deleted at least once (Fig 2B, top)—nevertheless, we again observe that paralogs are enriched, with increasing magnitude, among ever deleted (FET: OR = 1.24, P = 2e-5) and recurrently deleted passengers (FET: OR = 1.58, P = 7e-4; Fig 2B). To assess whether this

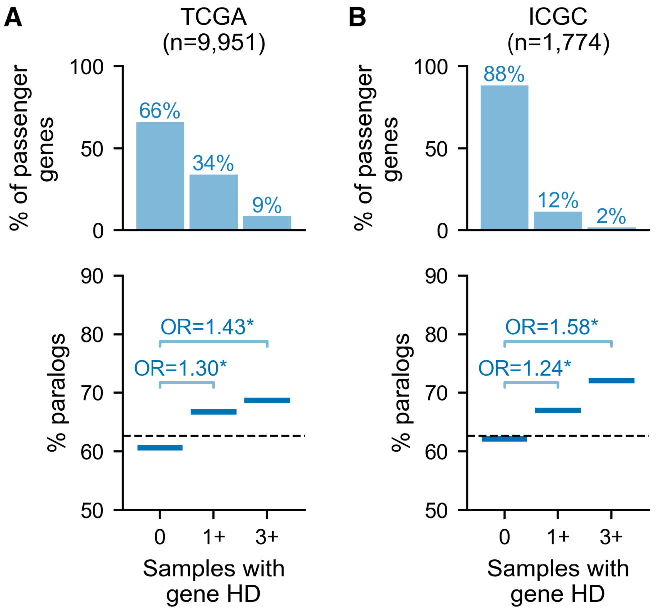

Figure 2. Homozygously deleted passenger genes are enriched in paralogs.

A Top: Percentage of passenger (non-driver) genes that are deleted in 0, at least 1 and at least 3 TCGA tumor samples. The number of TCGA tumor samples considered is shown. Bottom: For passenger genes grouped by their number of HDs, the percentage of genes in each group that are paralogs (Note: the 3+ group is a subset of the 1+ group). Annotations show the Odds Ratio (OR) for a Fisher's Exact Test comparing the percentage of paralogs among genes with 0 vs. 1+ HDs and 0 vs. 3+ HDs; asterisk (*) indicates $P < 0.05$. The dashed line shows the percentage of all passenger genes that are paralogs.

B Same as (A) but for ICGC tumor samples.

© 2023 The Authors

trend is also evident within tumors from individual cancer types, we separately analyzed each TCGA cancer type with at least 600 samples ($n = 9$). As the overall number of HDs is lower with the smaller sample sizes, we only compared genes that are never deleted vs. genes with at least one deletion in each sample subset. For eight out of nine cancer types, we observed that paralogs are significantly enriched among passengers with at least one HD (Fig EV4A, all $P < 0.05$ after Holm–Bonferroni correction). This observation indicates that the increased HD frequency of paralogs compared with singletons is robust to cancer type-specific copy number alteration biases, and to overall HD burden, which can vary considerably by cancer type (Zack *et al*, 2013; Cheng *et al*, 2017). For one cancer type (Colon Adenocarcinoma, COAD), the trend appears consistent, but the overall frequency of HDs is lower, and the enrichment is not statistically significant.

We next asked whether paralogs are also subject to more hemizygous deletions than singletons, which could lead to unequal rates of HD generation. To assess this, we identified all genomic segments with loss-of-heterozygosity (LOH), that is, all segments where one (but not both) of the alleles has copy number 0. We do not find that paralogs are more frequently subject to LOH than singletons in either the TCGA or ICGC cohort (Fig EV4B and C); when considering all LOH segments, we even see that singletons are slightly more frequently subject to LOH in the ICGC cohort (Fig EV4C, left), but when considering only focal LOH segments—that is, segments whose length is less than half of the chromosome arm's length, which is the case for all HD segments—there is no significant difference between paralog and singleton LOH frequency in either cohort. To assess whether gene dosage influenced the observed LOH frequency, we further restricted our analysis to copy neutral LOH events (total copy number = 2) and copy loss LOH events (total copy number = 1) and again found no significant increase in deletion frequency of paralogs compared with singletons (Fig EV4B and C). In strong contrast to what we saw for homozygous deletions, all passenger genes are hemizygously deleted in at least one tumor sample (in both cohorts), and most are frequently hemizygously deleted, underscoring that hemizygous gene loss appears to be well-tolerated (Van den Eynden *et al*, 2016; Martincorena *et al*, 2017). This broadly suggests that paralogs are not in general more prone to deletions than singletons—we investigate this further in the next section.

Paralogs are still more frequently homozygously deleted after accounting for proximity to tumor suppressors and fragile sites

Having established that HDs are more frequently observed for paralogs than singletons, we next wished to understand whether this could be attributed to other genome features, as copy number alterations of specific genomic regions can be influenced by multiple factors. In tumors, perhaps the most obvious influence is the presence of tumor suppressor genes (TSGs) whose loss may be subject to positive selection, driving up local deletion frequency (Zack *et al*, 2013; Cheng *et al*, 2017). However, there are additional factors that are known to influence copy number variation, including proximity to fragile sites, telomeres, and centromeres. Visual analysis of the observed gene HD frequency across the genome (Fig EV2) suggests that these factors are in some cases associated with higher HD frequency and thus they may explain the relatively higher HD frequency of paralogs—for example, perhaps paralogs are simply enriched in regions close to fragile sites. Here, we discuss each of the factors in turn, and then account for them collectively in our analysis.

First, paralogs may be more frequently homozygously deleted if they are in general located closer to recurrently deleted TSGs (Figs 3A and EV2). Typically, an HD results in the loss of several chromosomally adjacent genes— as noted, in the TCGA dataset a median of three genes are lost per gene-deleting HD segment. Genes adjacent to TSGs might therefore be deleted more frequently due to positive selection for the loss of the TSG. For example, multiple passenger genes adjacent to the recurrently deleted tumor suppressor *TGFBR2* are themselves also recurrently deleted, including *ZCWPW2* and *GADL1* (Fig 3A).

A second factor that could influence gene deletion frequency is proximity to fragile sites—chromosomal regions that are particularly prone to instability (Glover *et al*, 2017). Previous work has shown that some of the deletion hotspots in cancer genomes can be attributed to fragile sites (Bignell *et al*, 2010; Zack *et al*, 2013; Cheng *et al*, 2017). Genes close to fragile sites may thus be more likely to be deleted at least once in the evolutionary history of each tumor. To assess the impact of fragile sites, we obtained genomic coordinates for 15 major autosomal fragile sites identified in the PCAWG study (Li *et al*, 2020)—these sites were identified based on analyses of (a subset of) the TCGA and ICGC tumor samples used in this work. The fragile sites all overlap large (> 600 kb) genes, some of which, such as *FHIT* (Fig 3A), have also been identified as tumor suppressors—as was noted in Glover *et al* (2017). We observe that although HDs from the TCGA cohort do recur with relatively high frequency at these fragile sites, they are much shorter than other HD segments (mean length ~ 0.34 Mb vs. ~0.83 Mb) and are mainly intragenic, that is, they rarely result in even one full gene HD (Fig EV5A). However, given the low frequency of HDs overall, even small HD peaks, which can be observed over some fragile sites (Figs 3A and EV2), are important to take into account.

The third factor to consider is proximity to telomeres and centromeres, as (sub)telomeric and centromeric regions have been shown to be enriched for deletions both in cancer genomes specifically (Beroukhim *et al*, 2010; Zack *et al*, 2013; Cheng *et al*, 2017; Li *et al*, 2020), and across human genomes in general (Collins *et al*, 2020). As with fragile sites, HD generation could thus be higher for genes close to these regions – indeed, for several chromosomes we observe gene HD peaks at the chromosome edges or centromeres that are not explained by a known TSG or fragile site, e.g. at the start of chromosome 3 (Figs 3A and EV2).

To account for the influence of the potentially confounding factors described above, we first repeated our analysis of passenger HDs (from Fig 2A) after excluding all HD segments that at least partially delete a TSG, at least partially overlap a fragile site, or are telomere- or centromere-bound (see Materials and Methods). Figure 3A highlights the HD segments that are dropped among all HD segments on chromosome 3 that fully delete at least one passenger gene. In total, the excluded segments make up ~55% of all passenger-deleting HD segments in the TCGA data, which is mainly attributable to TSG-associated segments. As expected, when considering just the remaining HDs, the percentage of passenger genes with no HDs is higher, while the percentage of recurrently deleted passenger genes is lower (Fig 3B, top). Nevertheless, we find that

 © 2023 The Authors

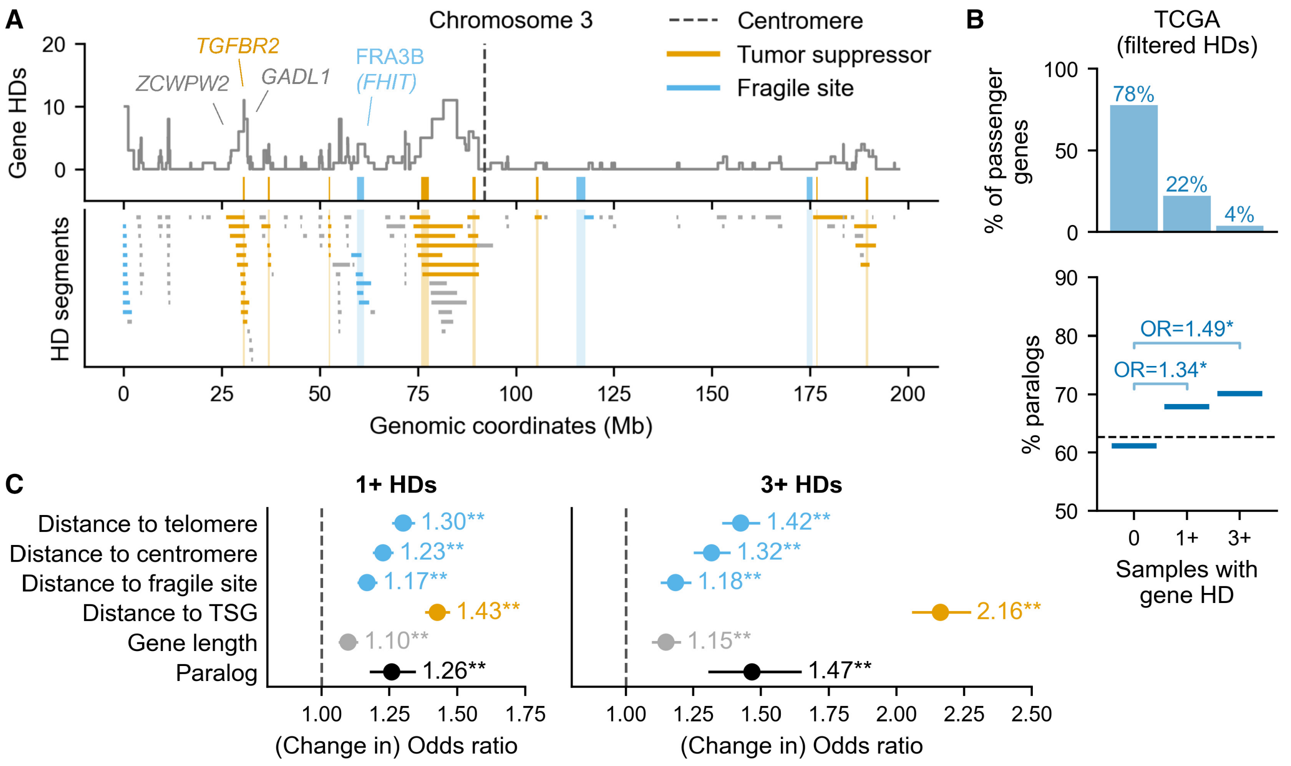

Figure 3. Having a paralog independently increases the odds of observing 1+ HDs for a passenger gene.

A Top: Line plot showing the number of TCGA samples in which each gene along chromosome 3 is homozygously deleted. Orange ticks show the location and width of TSGs that are deleted in at least 1 sample. Blue ticks show the location and width of fragile sites. Bottom: Location of all HD segments affecting chromosome 3. Segments are colored according to whether they overlap a TSG (orange) or a fragile site/telomere region (blue).

B Same as Fig 2A but calculated after excluding all segments that overlap a TSG, fragile site, telomere or centromere, i.e., the colored segments in (A).

C Odds ratio estimates for the association of paralogy and other genomic factors with observing 1+ HDs (left) and 3+ HDs (right) vs. 0 HDs of a passenger gene across tumor samples from TCGA and ICGC combined. Gene counts in each group as follows – 0 HDs (n = 10,453), 1+ HDs (n = 6,445) and 3+ HDs (n = 1,713). For all variables except paralogy the dot represents the change in odds ratio for a one standard deviation increase in the variable value. Lines indicate 95% confidence intervals and asterisks indicate P-values from the logistic regression (** = $P < 0.01$, * = $P < 0.05$).

paralogs are enriched among ever deleted passenger genes and even more enriched among recurrently deleted passenger genes (Fig 3B).

As fragile sites appear to be centered on large genes, and some additional recurrently deleted regions have been identified over large genes in tumor samples (Beroukhim *et al*, 2010; Glover *et al*, 2017), we considered that gene length (i.e., number of bases from the start of the first to end of the last exon for the longest transcript) might also be a potential confounder—particularly because we, and others (Ibn-Salem *et al*, 2017), observe that paralogs are on average significantly longer than singletons (mean gene length ~ 58 kb vs. ~39 kb, Mann–Whitney U (MWU) test, $P <$ 1e-16). However, as we only count full gene HDs, the impact of gene length on the likelihood of observing a gene HD is likely limited—the correlation between gene length and HD frequency is only 0.03 (Spearman's correlation coefficient, P = 6.5e-5), compared to 0.16 ($P <$ 1e-16) when counting all partial gene HDs.

To understand the relative contribution of paralogy and the other identified factors to HD frequency, we fit logistic regression models for ever and recurrent gene deletion (i.e., 0 vs. 1+ and 0 vs. 3+ HDs) that integrate paralogy, gene length (which we could not account for in Fig 3B) and distance to the nearest fragile site, telomere, centromere, and recurrently deleted TSG (Fig 3C and Dataset EV3; see Materials and Methods). The four distance variables are inverted so that higher values indicate the gene is closer to the region of interest and capped at 10 Mb—this corresponds to the ~99th percentile of all observed gene-deleting HD segment lengths and thus represents a plausible range of influence (Fig EV5B, see Materials and Methods). To increase statistical power for this regression analysis, we combine the TCGA and ICGC cohorts into one dataset by summing gene HDs. For the "distance to recurrently deleted TSG" variable, we thus consider distance to 90 TSGs that have at least three HDs across this joint dataset.

We find that paralogy is independently associated with higher odds of observing any HD for a passenger gene (OR = 1.26, Fig 3C), and that the increase in deletion odds associated with paralogy is greater for recurrent HDs (OR = 1.47)—corroborating what we observed earlier. For both the ever and recurrent HD models, the full model describes the data significantly better than a model that omits paralogy (likelihood ratio tests, $P <$ 1e-8). As might be expected, proximity to the nearest recurrently deleted TSG is the single most influential factor—a one standard deviation increase in this variable (which corresponds to ~1 Mb) increases the odds of observing any HD by a factor of 1.43 and the odds of observing recurrent HD by a factor of 2.16. The odds associated with the other

© 2023 The Authors

genomic factors are similar to or smaller than those associated with paralogy.

Overall, the logistic regression models for any and recurrent passenger gene HD suggest that having at least one paralog significantly increases the likelihood of observing at least one HD for a given gene, independently of the influence of positively selected TSG HDs, gene length or proximity to genomic fragile sites (including centromeres and telomeres). Being a paralog increases the odds of an HD ever being observed in the cohort by a factor of 1.26 and the odds of a recurrent HD in the cohort by a factor of 1.47.

Homozygous deletion frequency of paralog passengers is influenced by paralog properties

In previous work we, and others, observed that certain features of paralog genes make them more or less likely to be dispensable for the growth of cancer cell lines (Dandage & Landry, 2019; De Kegel & Ryan, 2019). Specifically, we found that genes from larger paralog families and genes with a more sequence similar paralog are more likely to be dispensable—presumably because those properties can be linked to increased buffering capacity for paralog loss. We also found that paralogs that originated from a whole genome duplication (WGD), as opposed to a small scale duplication (SSD), were both more likely to be essential at least some of the time, but less likely to be essential all of the time—this could be explained by WGD paralogs being more likely to be synthetic lethal (De Kegel & Ryan, 2019; De Kegel *et al*, 2021). Having determined that paralog passenger genes are overall more likely to be homozygously deleted in tumors, we next asked whether there are differences in the deletion frequency of paralogs with different properties.

To answer this question, we first compared family size for paralog passenger genes grouped according to whether they are ever and/or recurrently deleted. We find that paralog genes with at least one HD across tumor samples from both cohorts are more likely to come from a big paralog family, that is, have more than the median number of paralogs (3), than those that are never deleted (FET: OR = 1.25, P = 5e-8; Fig 4A). Furthermore, the odds of belonging to a big family increase for recurrently deleted paralogs (FET: OR = 1.42, P = 6e-8). This is in line with what was previously observed for paralog dispensability in cancer cell lines.

We next compared, for the same groups of paralog passengers, the sequence identity each gene shares with its closest (i.e., most sequence identical) paralog (Fig 4B). We observe that, on average, sequence identity is slightly higher for passengers with at least one HD compared to those with no HDs (mean ~ 52% vs. ~54%, MWU test: P = 2e-6), and higher again for passengers with recurrent HD (mean ~ 56%, MWU test for 0 vs. 3+ HDs: P = 3e-9). This is again consistent with what we observed in cancer cell lines and suggests that passenger genes with a potentially more functionally similar paralog are more likely to be dispensable.

Third, we asked whether we could observe differences in the HD frequency of WGD vs. SSD paralogs—we annotate individual paralog genes as WGD if they are part of at least one WGD pair, and as SSD otherwise. As WGD paralogs are more likely to be conditionally essential than SSD paralogs there is no clear expectation, based on what we saw in cancer cell lines, for whether the loss of an individual WGD gene will be more or less dispensable. We find that passengers with at least one HD are depleted in WGDs compared to paralogs with no HDs (FET: OR = 0.87, P = 0.0005) and that passengers with recurrent HD are further depleted in WGDs (FET: 0 vs. 3+ HDs: OR = 0.79, P = 0.0002, Fig 4C); thus, WGD paralogs appear to be less dispensable for tumors than SSD paralogs.

To assess whether family size, sequence identity and duplication mode (WGD vs. SSD) independently influence the probability of observing a passenger gene HD—given that these properties are to some extent correlated—we fitted similar logistic regression models to the ones described in the previous section. However, this time, as we are interested in understanding variation among paralogs, we restricted our analysis to paralog genes (i.e., excluded singletons) and added terms for each of the three paralog properties (see Materials and Methods). We find that, to varying extents, each paralog property significantly affects the odds of observing any HD, and that the magnitude of the odds ratios associated with each property increases when considering recurrent HD (Fig 4D). Considering the 1+ HD model, we find that the full model fits the data significantly better than models which omit one of the three paralog properties (likelihood ratio tests for full model vs. model without: WGD, P = 0.008; big family, P = 0.0003; sequence identity, P = 0.0004)—suggesting that each paralog property has a significant independent influence on paralog HD frequency.

Essential paralogs are less frequently homozygously deleted than non-essential paralogs

While paralogs are in general more dispensable, certain paralogs are instead essential for cellular growth. If the key factor contributing to the increased frequency of paralog vs. singleton HDs is paralog dispensability, we would expect to see that essential paralogs are deleted less frequently than non-essential paralogs. Such an observation would indicate that higher homozygous deletion frequency is not a blanket property of paralog genes, but rather tied to their dispensability.

To estimate gene essentiality, we first use a list of broadly essential genes identified from genome-wide CRISPR screens in 769 cancer cell lines (De Kegel *et al*, 2021). These are genes that are associated with a substantial reduction in cellular growth in at least 90% of screened cell lines (see Materials and Methods). Grouping paralog passenger genes according to whether they are ever and/or recurrently deleted in the TCGA cohort (similar to Fig 2A, but considering paralog genes only), we find that essential paralogs are significantly depleted among paralog passengers with any HD (Fig 5A, top; OR = 0.42, P = 6e-7), and additionally depleted among paralog passengers with recurrent HD (OR = 0.14, P = 3e-6). Thus, while HDs are more likely to be observed across tumor samples for paralogs as a whole, they are less likely to be observed for cell-essential paralogs.

We confirmed this tendency using two other sets of essential genes: the 1,734 genes identified as essential from gene trap screens performed in KMB7 and HAP1 cell lines (Blomen *et al*, 2015) (Fig 5B, top); and the overlap of the top 2000 negative hits from genome-wide CRISPR screens in three lung cancer cell lines grown in 3D spheroids (Han *et al*, 2020) (Fig 5C, top; see Materials and Methods). Although growth phenotypes *in vitro*, even in 3D, do not perfectly recapitulate such phenotypes *in vivo*, the consistently strong pattern for each of the three essentiality datasets suggests that they provide a reasonable approximation. We again validated

 © 2023 The Authors

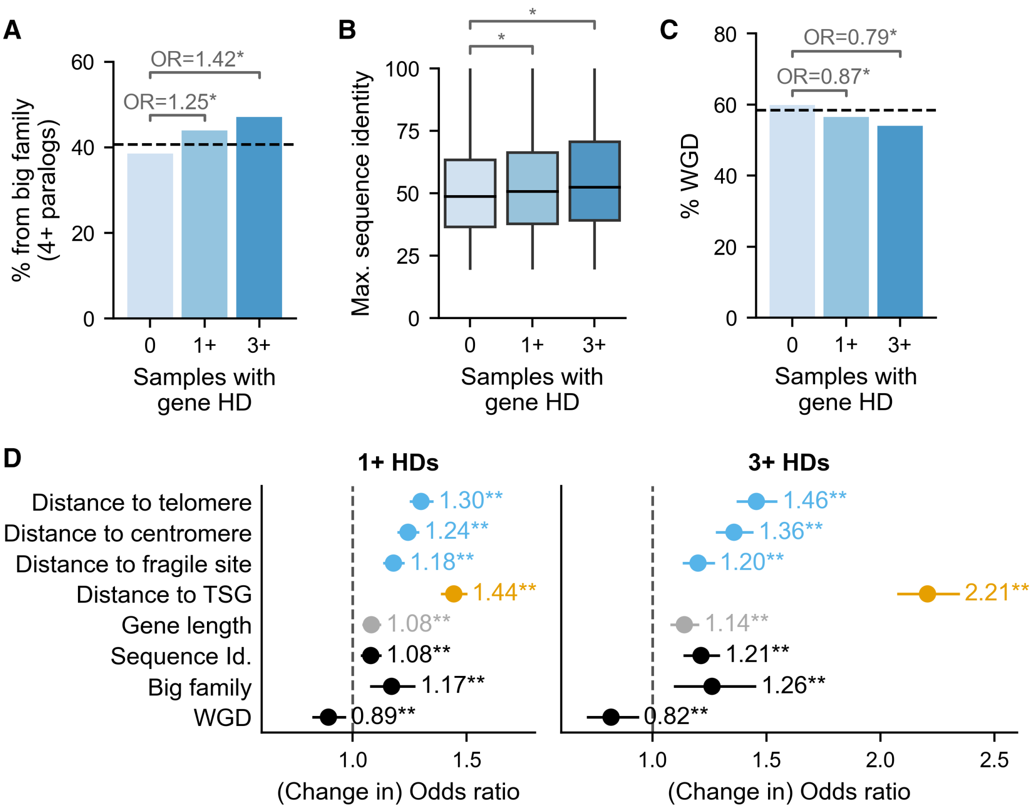

Figure 4. Paralog properties correlate with HD frequency.

A Bar plot showing the percentage of paralog genes that are part of a big paralog family (i.e. have at least 4 paralogs) among paralog passengers with 0, 1+ or 3+ HDs across TCGA and ICGC samples. Annotations show the Odds Ratio (OR) from Fisher's Exact Tests comparing paralogs with 0 vs. 1+ HDs and 0 vs. 3+ HDs; asterisk (*) indicates $P < 0.05$.

B Box plots showing the maximum sequence identity, i.e. sequence identity with the closest paralog, for paralog passengers with 0, 1+ or 3+ HDs; asterisk (*) indicates $P < 0.05$, MWU test. The boxes represent the first and third quartiles (Q1 and Q3) of the distribution, the horizontal black line the median, and the whiskers extend up to 1.5*the interquartile range past Q1 and Q3.

C Similar to (A): bar plot showing the percentage of whole genome duplicates (WGDs) among paralog passenger genes with 0, 1+ or 3+ HDs. Annotations show the Odds Ratio (OR) from Fisher's Exact Tests comparing paralogs with 0 vs. 1+ HDs and 0 vs. 3+ HDs; asterisk (*) indicates $P < 0.05$.

D Similar to Fig 3C: Odds ratio estimates for the association of sequence identity, big paralog family, WGD and other genomic features with observing 1+ HDs (left) and 3+ HDs (right) vs. 0 HDs of a paralog passenger gene across tumor samples from TCGA and ICGC. For all variables except 'WGD' and 'Big family', which are Boolean, the dot represents the change in odds ratio for a one standard deviation increase in the variable value. Lines indicate 95% confidence intervals and asterisks indicate *P*-values from the logistic regression (** = $P < 0.01$, * = $P < 0.05$). Gene counts in each group as follows – 0 HDs (n = 6,335), 1+ HDs (n = 4,253) and 3+ HDs (n = 1,179).

our findings with tumor samples from the ICGC cohort (Fig 5, bottom). Overall, this suggests that the key reason for the increased HD frequency of paralogs vs. singletons is paralog dispensability.

Discussion

In this work we found that, across large patient cohorts, homozygous deletions are more likely to be observed for paralog than singleton non-driver genes. The influence of paralogy on HD frequency is independent of factors that may increase genomic copy number variation, such as proximity to frequently deleted TSGs or fragile sites. In addition, we found that properties of paralogs that are associated with increased dispensability in cancer cell lines are also associated with increased HD frequency across tumor genomes. Finally, we found that HDs are less likely to be observed for essential compared to non-essential paralogs—thus, rather than being a blanket property of paralog genes, increased HD frequency appears to be tied to gene essentiality. Overall we conclude that HDs are more likely to be observed for paralogs because paralogs are on average more dispensable and their loss is thus not selected against as strongly as singleton loss.

Most previous studies of negative selection in cancer used some form of normalized dN/dS ratio to quantify selection strength and found that, in contrast to positive selection, the signal of negative selection appears to be largely absent, outside of inactivating mutations in essential genes in haploid regions (Van den Eynden *et al*, 2016; Bakhoum & Landau, 2017; Martincorena *et al*, 2017; Weghorn & Sunyaev, 2017; López *et al*, 2020). One potential reason for this is that, given current sample sizes, there is lower statistical power to detect a significant depletion of aberrations in comparison with a significant enrichment (Weghorn &

© 2023 The Authors

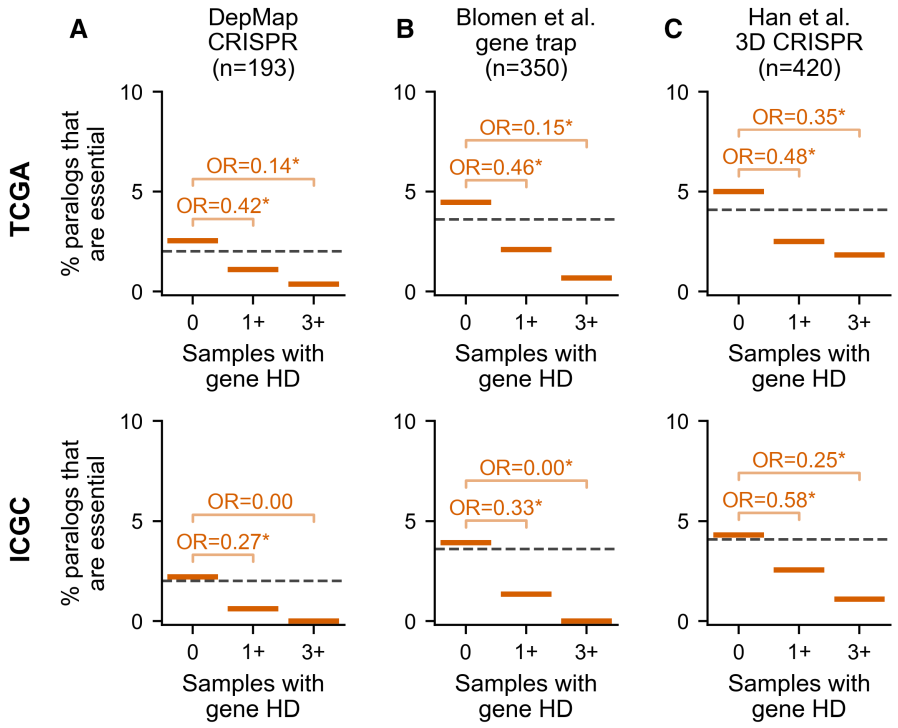

Figure 5. Essential paralog passengers are less likely to be homozygously deleted than non-essential paralog passengers.

A–C The percentage of paralog genes that are essential, according to dependency data from DepMap CRISPR screens, among paralog passengers with 0, 1+ or 3+ HDs in the TCGA (top) or ICGC (bottom) cohort. Annotations show the Odds Ratio (OR) from Fisher's Exact Tests comparing essential paralogs with 0 vs. 1+ HDs and 0 vs. 3+ HDs; asterisk (*) indicates $P < 0.05$. The total number of essential paralog passengers identified from the DepMap CRISPR screens is shown; the percentages are calculated in reference to all paralog passengers for which DepMap essentiality data was available. Essential genes for the DepMap dataset (Meyers *et al*, 2017) are obtained from a version of the data reprocessed by De Kegel *et al* (2021) to reduce off-target sgRNA effects (see Materials and Methods) (B) Same as (A) but for essentiality data from Blomen *et al* gene trap screens (C) Same as (A) but for essentiality data from Han *et al* 3D CRISPR screens.

Sunyaev, 2017; Zapata *et al*, 2018). To address this limitation, we focused on HD frequency as a blunt tool for assessing the relative strength of negative selection acting on gene loss. We anticipated that negative selection might be more apparent for HDs, which result in complete protein loss, than for non-synonymous mutations, particularly as many genes essential for cellular growth appear to be haplo-sufficient (Wang *et al*, 2015; Van den Eynden *et al*, 2016). While our approach does not allow us to pinpoint specific cases where gene loss is selected against, we could show that in general deletion of paralogs appears to be under weaker negative selective pressure. As paralogs make up over 60% of protein-coding genes, we propose that their increased dispensability could also in part explain the low level of negative selection that has been previously observed using dN/dS approaches (Martincorena *et al*, 2017; Weghorn & Sunyaev, 2017).

The impact of negative selection could also appear to be limited if weakly deleterious mutations are not weeded out—this can occur when, due to the lack of recombination during (asexual) tumor cell reproduction, deleterious mutations are co-inherited with alterations that confer a strong fitness advantage (Tilk *et al*, 2022). We observed that proximity to a frequently deleted TSG has a strong impact on HD likelihood for non-driver genes (Fig 3C), but it is unclear whether this excludes influence from negative selection. It is possible that the observed TSG-targeting HDs were retained in part due to the dispensability of the co-deleted genes (McFarland *et al*, 2014). In support of this hypothesis, Pertesi *et al* (2019) showed that, for several TSGs, the frequency of passenger HDs decreases approximately linearly between the tumor suppressor and the nearest essential genes on either side.

Much of our understanding of the factors that influence gene dispensability comes from studies in model organisms, in particular the budding yeast *Saccharomyces cerevisiae* (Gu *et al*, 2003; Guan *et al*, 2007; Hakes *et al*, 2007; Dean *et al*, 2008; DeLuna *et al*, 2008). Analyses of the yeast gene deletion collection, a set of gene deletion mutants systematically generated in a single *S. cerevisiae* strain, revealed that paralogs were less likely to be essential than singleton genes (Giaever *et al*, 2002; Gu *et al*, 2003). Furthermore, more detailed analyses of yeast paralogs revealed that paralogs from large families were less likely to be essential and so were genes with highly sequence similar paralogs (Guan *et al*, 2007; Hakes *et al*, 2007). Previous analyses, including our own, demonstrated that many of these trends are also evident when analyzing gene essentiality from CRISPR screens in cancer cell lines (Blomen *et al*, 2015; Dandage & Landry, 2019; De Kegel & Ryan, 2019; Dede *et al*, 2020). Our results here are also consistent with these findings—many of the features that are associated with paralog dispensability in yeast are also associated with gene deletion frequency in tumor genomes.

The connection between the budding yeast observations and those in cancer is less clear when it comes to the relative dispensability of WGDs and SSDs. Analyses of the yeast gene deletion collection revealed that SSDs are more likely to be essential than

 © 2023 The Authors

WGDs in the single genetic background studied (Guan *et al*, 2007; Hakes *et al*, 2007). In our previous analyses of gene essentiality in hundreds of cancer cell lines, we found that SSDs were more likely to be *broadly* essential (essential in most cell lines) than WGDs but that WGDs were less likely to be *never* essential (i.e., more likely to be essential in at least one cell line) (De Kegel & Ryan, 2019). As the analyses of gene essentiality in budding yeast were generated in a single genetic background the concordance with our cancer cell line results was difficult to assess, but as gene deletion collections are now being generated in additional yeast strains, it should become possible to perform a more direct comparison (Galardini *et al*, 2019; Caudal *et al*, 2022; Wang *et al*, 2022).

Here, we found that WGDs are less likely to be deleted than SSDs in tumors. This is surprising in light of the yeast gene deletion collection results, where SSDs were more likely to be essential than WGDs in the strain studied, but less so in light of the cancer cell line results, where WGDs were less likely to be *never* essential. It is also worth noting that experimental evolution studies in yeast found that SSDs accumulate protein-altering mutations at a higher rate than WGDs (Fares *et al*, 2013; Keane *et al*, 2014). These results are perhaps especially relevant when analyzing the influence of paralog features on selection in tumors.

We note that there are many additional differences in the features of WGDs and SSDs in budding yeast that may alter their relative dispensability in tumors. An obvious large-scale difference is that in the ancestor of humans there were two rounds of whole genome duplication compared to a single duplication event in yeast (Wolfe & Shields, 1997; Dehal & Boore, 2005). Less obvious, but potentially of importance for cancer, is that the two classes of paralogs are enriched in pathways in humans that do not have obvious counterparts in yeast. For example, WGDs are highly enriched in signaling pathways involved in development while SSDs are enriched in immune response genes (Huminiecki & Heldin, 2010). How the membership of these pathways influences the dispensability and selection of genes in tumors and cancer cell lines warrants further study.

In both yeast and cancer, there are a number of reasons for why paralogs might be more dispensable than singleton genes. Perhaps the most obvious is the existence of buffering relationships between paralog pairs, such that when one paralog is lost the other paralog can compensate for this loss. Such buffering relationships between paralogs can be revealed through synthetic lethality screens and a number of recurrently deleted paralogs in cancer have already been reported to display synthetic lethal interactions with their paralog (recently reviewed in Ryan *et al* 2023). Supporting this model, in previous work analyzing essentiality in cancer cell lines we found that buffering relationships between paralogs could explain 13–17% of cases where a paralog was essential in some cell lines but not others (De Kegel & Ryan, 2019). This suggests that at least some of the increased dispensability of paralogs in cancer cells can be attributed to buffering relationships between paralog pairs. However this is not the only explanation for paralogs displaying increased dispensability in tumor cells. An additional explanation is that paralogs may perform essential functions in specific contexts (e.g., within specific tissues or at specific developmental stages) but are not required within the specific context of a tumor. Consistent with this model, human paralogs are more likely to display tissue-specific expression patterns (Huminiecki & Wolfe, 2004). Finally we note that there is evidence to suggest that genes whose perturbation has a lower phenotypic impact may be more "duplicable"—that is, rather than paralogs being under weaker selection because they are duplicated, their duplication was tolerated because they were already under weaker selection (He & Zhang, 2006; O'Toole *et al*, 2018). Teasing apart the relative contributions of these factors to the increased dispensability of paralogs in cancer will require further research and potentially new data resources such as gene essentiality profiles in diverse non-cancer cell types (Tian *et al*, 2021). Analyzing the frequency with which two members of a paralog family are lost would provide more direct insight into the contribution of paralog redundancy, but, due to the overall rarity of passenger gene HDs, we cannot make a comprehensive assessment of co-deletions here—for example, among paralog pairs where both genes are non-drivers, and not on the same chromosome, only two pairs are co-deleted in at least one TCGA sample. Larger cohorts would allow us to search for patterns of mutual exclusivity between HDs of paralog genes to identify genetic interactions—this has been applied for identifying interactions between driver genes (Ciriello *et al*, 2012; Canisius *et al*, 2016), but is more challenging for interactions between non-driver genes, which are much less frequently altered.

One of the potential means of targeting gene loss in cancer is through the identification of synthetic lethal interactions. Recent systematic efforts have focused on identifying synthetic lethal interactions involving paralog pairs in cancer cell lines (Dede *et al*, 2020; De Kegel *et al*, 2021; Parrish *et al*, 2021; Thompson *et al*, 2021). These interactions can potentially be exploited to develop targeted therapies that selectively kill tumor cells with recurrently deleted paralogs (Muller *et al*, 2012; D'Antonio *et al*, 2013; Oike *et al*, 2014; Lord *et al*, 2020). Our results provide an explanation for why the loss of paralogs is so common in tumor genomes and hence why such approaches may be broadly applicable.

One potential limitation of our analysis is that some of the driver and non-driver genes may be misclassified. For instance, it is likely that there are some frequently deleted TSGs in the patient cohorts that are not currently included in the Cancer Gene Census or identified by Bailey *et al* (2018)—although the latter analyzed the TCGA cohort specifically, TSGs were mainly identified from mutation rather than copy number data. However, given that we analyzed all non-driver genes collectively, it is unlikely that our conclusions would be altered significantly by the addition or removal of a small number of driver annotations. A second limitation, indicated by our saturation analysis (Fig EV3), is that there are likely many gene HDs that are unobserved due to limited sample size, rather than negative selection. As larger patient cohorts are assembled, it will become possible to dissect further the influences of paralogy and other factors—for example, membership of specific pathways—on HD frequency.

Materials and Methods

Identifying HDs in TCGA tumor samples

We initially obtained 9,966 copy number profiles for tumor samples from the Cancer Genome Atlas (Cancer Genome Atlas Research Network *et al*, 2013) that had already been processed with ASCAT (Van Loo *et al*, 2010); downloaded from https://github.com/VanLoo-lab/ascat/tree/master/ReleasedData/TCGA_SNP6_hg19.

© 2023 The Authors

The ASCAT results were filtered to those for which QC= = "Pass." We straightforwardly identified HD segments as those where both the major and minor allele copy number is 0. To map genes to HD segments, we used the annotations from the consensus coding sequence database (GRCh37.p13 assembly, CCDS release 15) (Pujar *et al*, 2018) and considered a gene to be deleted if its coding region is fully within the bounds of an HD segment. We consider a gene's coding region to range from the start of the first exon to the end of the last exon of its longest transcript. Six genes are fully or partially outside the genomic region mapped by the SNP6 microarray, so we exclude these from analysis. We dropped 15 "hyper-deleted" samples with over 100 homozygously deleted genes from our analysis as this far exceeds the number of deleted genes in the majority of samples (Fig EV1A). The remaining 9,951 samples were used for all analyses.

Identifying HDs in ICGC tumor samples

We use the 1,782 white-listed ICGC samples from the PCAWG study (ICGC/TCGA Pan-Cancer Analysis of Whole Genomes Consortium, 2020). Although the PCAWG study included both ICGC and TCGA samples, we only use the ICGC samples to ensure that this constitutes an independent dataset. The segment-level allele-specific copy number calls in PCAWG are based on the consensus of six algorithms applied to whole genome sequencing data. We then followed the same procedure as above for identifying homozygous deletions, and in this case dropped eight "hyper-deleted" samples with over 100 deleted genes—these again represent extreme outlier samples (Fig EV1B).

Driver genes

We compiled a broad list of 652 driver genes from the Cancer Gene Census (CGC) (Sondka *et al*, 2018) and Bailey *et al* (2018), including both Tier 1 and 2 entries from the CGC. We considered genes to be potential TSGs if either: (1) the "Role in Cancer" field in the CGC dataset included "TSG" or was blank; or (2) the gene was labeled as "tsg" or "likely tsg," or was "rescued," in the Bailey et al dataset. This gave us a list of 411 TSGs from which we identified (recurrently) deleted TSGs for the analyses related to Figs 3 and 4.

Identifying and annotating passenger genes

We started with a comprehensive list of autosomal protein-coding genes from the HGNC (Braschi *et al*, 2018) (2021-07-01 release), and filtered this down to genes for which there are annotations in the hg19 version of the CCDS. From this list, we then filtered out the driver genes described above as well as 16 genes whose coding regions, according to CCDS, at least partially overlap the coding region of one of those driver genes. A gene was annotated as a paralog if it has at least one paralog in Ensembl 93 with which it shares at least 20% reciprocal sequence identity (Zerbino *et al*, 2018). Maximum sequence identity for each paralog gene was also obtained from Ensembl 93. We labeled paralog pairs as whole genome duplicates (WGDs) based on their inclusion in the list of WGDs identified by Makino and McLysaght (2010) or the strict list of WGDs in the OHNOLOGS v2 resource (Singh & Isambert, 2020). All genes that are part of at least one such WGD pair are labeled as WGD, while the rest are labeled as SSD.

Identifying centromere and telomere-bound HDs in the TCGA cohort

As the copy number profiles for the TCGA data are computed from SNP6 array data, the genomic regions for which copy number is available are limited by the coverage of the array probes. Thus, to identify likely centromere- and telomere-bound HDs we identified the boundaries, in terms of genomic coordinates, of the data output by ASCAT. We consider a segment to be telomere-bound if it starts or ends at the outer boundaries, that is, the first or last seen genomic coordinates respectively. Similarly, we consider a segment to be centromere-bound if it ends/starts at the maximum/minimum observed coordinate before/after the centromere assembly gap in hg19/GRCh37. Coordinates of telomere and centromere assembly gaps were obtained from the UCSC Genome Browser.

Logistic regression models for 1+ and 3+ passenger HDs

For all passenger genes, we fit logistic regression models with this form:

$$n + \text{HDs} \sim \text{Paralog} + \text{Gene length} + \text{Dist. to TSG} + \text{Dist. to fragile site} \\ + \text{Dist. to centromere} + \text{Dist. to telomere.}$$

Where the model variables are as follows:

- *n+ HDs* is a Boolean variable describing whether a gene has 1+ or 3+ (vs. 0) HDs across both cohorts (TCGA and ICGC).
- *Paralog* is a Boolean variable describing whether the gene has at least one paralog.
- *Gene length* is the number of bases from the start of the first exon to the end of the last exon for the longest transcript associated with the gene (z-scored).
- *Dist. to TSG* is the inverted distance from the gene to the nearest recurrently deleted (i.e., 3+ HDs) TSG (z-scored). We cap the distance at which a TSG could influence passenger deletion at 10 Mb, as this corresponds to the ~99th percentile of observed HD segment lengths across both cohorts, and then use 10 minus distance as the variable's value. Thus, a value of 10 implies the gene is right next to a recurrently deleted TSG, and a value of 0 implies the gene is > 10 Mb from a recurrently deleted TSG, or on a chromosome without a recurrently deleted TSG.
- *Dist. to fragile site* is the inverted distance from the gene to the nearest fragile site (z-scored). Distance is computed as for *Dist. to TSG*.
- *Dist. to telomere* and *Dist. to centromere* are the inverted distances from the gene to the nearest telomere and centromere respectively (z-scored). These distances are also computed as for *Dist. to TSG*.

For paralog passenger genes only, we fit logistic regression models with this form:

$$n + \text{HDs} \sim \text{WGD} + \text{Big family} + \text{Sequence Id.} + \text{Gene length} \\ + \text{Dist. to TSG} + \text{Dist. to fragile site} \\ + \text{Dist. to centromere} + \text{Dist. to telomere.}$$

© 2023 The Authors

Where the new model variables (not described above) are as follows:

- *WGD* is a Boolean variable that indicates whether the gene is part of a paralog pair that originated with a whole genome duplication.
- *Big family* is a Boolean variable that indicates whether the gene has at least four paralogs with which it shares at least 20% reciprocal sequence identity.
- *Sequence Id.* is the sequence identity the gene shares with its closest paralog, that is, its most sequence identical paralog (z-scored).

To fit these models, we used the "Logit" function from the python statsmodels package (version 0.11.0) (Seabold & Perktold, 2010) with default parameters.

Identifying essential genes

To identify broadly essential genes from the DepMap CRISPR screens, we used the gene dependency scores from De Kegel *et al* (2021). These gene dependency scores were computed with CERES (Meyers *et al*, 2017) after filtering out potential multi-targeting single guide RNAs, which disproportionately affect paralog dependency scores. Broadly essential genes were identified as those with CERES score < -0.6 in at least 90% of cell lines ($n = 969$). Essential genes from the Blomen et al study ($n = 1{,}664$) are those that were identified as essential in both the KBM7 and HAP1 cell lines (Blomen *et al*, 2015). Finally essential genes from the Han et al study ($n = 1{,}429$) are those that fall within the top 2000 (based on T-score) significant negative hits in at least two out of the three cell lines screened (Han *et al*, 2020). The numbers of genes indicated refer to the number of (autosomal) essential genes after merging with our full gene list.

Data availability

The processed and filtered homozygous gene deletion tables generated as part of this study are available as Dataset EV1 (TCGA) and Dataset EV2 (ICGC). The gene deletion frequency table with features used in the logistic regression analyses is available in Dataset EV3. Data analysis was performed using Python version 3.7, Statsmodels 0.11.0 (Seabold & Perktold, 2010) and Pandas 1.0.2 (McKinney, 2011). Notebooks with the code for all figures and statistical analysis are available at: https://github.com/cancergenetics/paralog_HDs.

Expanded View for this article is available online.

Acknowledgements
We thank the Peter van Loo lab for making the ASCAT v3 profiles of the TCGA samples publicly available, and Dr. Tom Lesluyes for answering questions regarding this data. We thank Dr. Rory Johnson, Dr. David Adams, Prof. Kenneth Wolfe, and members of the Ryan lab for providing helpful feedback on the manuscript. The results published here are in whole or part based upon data generated by the TCGA Research Network: https://www.cancer.gov/tcga. BDK and CJR were funded through an Irish Research Council 2017/2018 Laureate Award awarded to CJR. CJR is also supported by Science Foundation Ireland under grant number 20/FFP-P/8641.

Author contributions
Barbara De Kegel: Conceptualization; data curation; formal analysis; visualization; writing – original draft; writing – review and editing. **Colm J Ryan:** Conceptualization; formal analysis; supervision; funding acquisition; writing – original draft; writing – review and editing.

Disclosure and competing interests statement
The authors declare that they have no conflict of interest.

References

Bailey MH, Tokheim C, Porta-Pardo E, Sengupta S, Bertrand D, Weerasinghe A, Colaprico A, Wendl MC, Kim J, Reardon B *et al* (2018) Comprehensive characterization of cancer driver genes and mutations. *Cell* 173: 371–385

Bakhoum SF, Landau DA (2017) Cancer evolution: no room for negative selection. *Cell* 171: 987–989

Beroukhim R, Mermel CH, Porter D, Wei G, Raychaudhuri S, Donovan J, Barretina J, Boehm JS, Dobson J, Urashima M *et al* (2010) The landscape of somatic copy-number alteration across human cancers. *Nature* 463: 899–905

Bignell GR, Greenman CD, Davies H, Butler AP, Edkins S, Andrews JM, Buck G, Chen L, Beare D, Latimer C *et al* (2010) Signatures of mutation and selection in the cancer genome. *Nature* 463: 893–898

Blomen VA, Májek P, Jae LT, Bigenzahn JW, Nieuwenhuis J, Staring J, Sacco R, van Diemen FR, Olk N, Stukalov A *et al* (2015) Gene essentiality and synthetic lethality in haploid human cells. *Science* 350: 1092–1096

Braschi B, Denny P, Gray K, Jones T, Seal R, Tweedie S, Yates B, Bruford E (2018) Genenames.org: the HGNC and VGNC resources in 2019. *Nucleic Acids Res* 47: D786–D792

Cancer Genome Atlas Research Network, Weinstein JN, Collisson EA, Mills GB, Shaw KRM, Ozenberger BA, Ellrott K, Shmulevich I, Sander C, Stuart JM (2013) The Cancer Genome Atlas Pan-Cancer analysis project. *Nat Genet* 45: 1113–1120

Canisius S, Martens JWM, Wessels LFA (2016) A novel independence test for somatic alterations in cancer shows that biology drives mutual exclusivity but chance explains most co-occurrence. *Genome Biol* 17: 261

Caudal E, Friedrich A, Jallet A, Garin M, Hou J, Schacherer J (2022) Loss-of-function mutation survey revealed that genes with background-dependent fitness are rare and functionally related in yeast. *Proc Natl Acad Sci USA* 119: e2204206119

Cheng J, Demeulemeester J, Wedge DC, Vollan HKM, Pitt JJ, Russnes HG, Pandey BP, Nilsen G, Nord S, Bignell GR *et al* (2017) Pan-cancer analysis of homozygous deletions in primary tumours uncovers rare tumour suppressors. *Nat Commun* 8: 1221

Ciriello G, Cerami E, Sander C, Schultz N (2012) Mutual exclusivity analysis identifies oncogenic network modules. *Genome Res* 22: 398–406

Collins RL, Brand H, Karczewski KJ, Zhao X, Alföldi J, Francioli LC, Khera AV, Lowther C, Gauthier LD, Wang H *et al* (2020) A structural variation reference for medical and population genetics. *Nature* 581: 444–451

Dandage R, Landry CR (2019) Paralog dependency indirectly affects the robustness of human cells. *Mol Syst Biol* 15: e8871

D'Antonio M, Guerra RF, Cereda M, Marchesi S, Montani F, Nicassio F, Di Fiore PP, Ciccarelli FD (2013) Recessive cancer genes engage in negative genetic interactions with their functional paralogs. *Cell Rep* 5: 1519–1526

De Kegel B, Ryan CJ (2019) Paralog buffering contributes to the variable essentiality of genes in cancer cell lines. *PLoS Genet* 15: e1008466

© 2023 The Authors

De Kegel B, Quinn N, Thompson NA, Adams DJ, Ryan CJ (2021) Comprehensive prediction of robust synthetic lethality between paralog pairs in cancer cell lines. *Cell Syst* 12: 1144–1159

Dean EJ, Davis JC, Davis RW, Petrov DA (2008) Pervasive and persistent redundancy among duplicated genes in yeast. *PLoS Genet* 4: e1000113

Dede M, McLaughlin M, Kim E, Hart T (2020) Multiplex enCas12a screens detect functional buffering among paralogs otherwise masked in monogenic Cas9 knockout screens. *Genome Biol* 21: 262

Dehal P, Boore JL (2005) Two rounds of whole genome duplication in the ancestral vertebrate. *PLoS Biol* 3: e314

DeLuna A, Vetsigian K, Shoresh N, Hegreness M, Colón-González M, Chao S, Kishony R (2008) Exposing the fitness contribution of duplicated genes. *Nat Genet* 40: 676–681

Fares MA, Keane OM, Toft C, Carretero-Paulet L, Jones GW (2013) The roles of whole-genome and small-scale duplications in the functional specialization of Saccharomyces cerevisiae genes. *PLoS Genet* 9: e1003176

Galardini M, Busby BP, Vieitez C, Dunham AS, Typas A, Beltrao P (2019) The impact of the genetic background on gene deletion phenotypes in Saccharomyces cerevisiae. *Mol Syst Biol* 15: e8831

Giaever G, Chu AM, Ni L, Connelly C, Riles L, Véronneau S, Dow S, Lucau-Danila A, Anderson K, André B *et al* (2002) Functional profiling of the *Saccharomyces cerevisiae* genome. *Nature* 418: 387–391

Glover TW, Wilson TE, Arlt MF (2017) Fragile sites in cancer: more than meets the eye. *Nat Rev Cancer* 17: 489–501

Gu Z, Steinmetz LM, Gu X, Scharfe C, Davis RW, Li W-H (2003) Role of duplicate genes in genetic robustness against null mutations. *Nature* 421: 63–66

Guan Y, Dunham MJ, Troyanskaya OG (2007) Functional analysis of gene duplications in *Saccharomyces cerevisiae*. *Genetics* 175: 933–943

Hakes L, Pinney JW, Lovell SC, Oliver SG, Robertson DL (2007) All duplicates are not equal: the difference between small-scale and genome duplication. *Genome Biol* 8: R209

Han K, Pierce SE, Li A, Spees K, Anderson GR, Seoane JA, Lo Y-H, Dubreuil M, Olivas M, Kamber RA *et al* (2020) CRISPR screens in cancer spheroids identify 3D growth-specific vulnerabilities. *Nature* 580: 136–141

He X, Zhang J (2006) Higher duplicability of less important genes in yeast genomes. *Mol Biol Evol* 23: 144–151

Huminiecki L, Heldin CH (2010) 2R and remodeling of vertebrate signal transduction engine. *BMC Biol* 8: 146

Huminiecki L, Wolfe KH (2004) Divergence of spatial gene expression profiles following species-specific gene duplications in human and mouse. *Genome Res* 14: 1870–1879

Ibn-Salem J, Muro EM, Andrade-Navarro MA (2017) Co-regulation of paralog genes in the three-dimensional chromatin architecture. *Nucleic Acids Res* 45: 81–91

ICGC/TCGA Pan-Cancer Analysis of Whole Genomes Consortium (2020) Pan-cancer analysis of whole genomes. *Nature* 578: 82–93

Kamath RS, Fraser AG, Dong Y, Poulin G, Durbin R, Gotta M, Kanapin A, Le Bot N, Moreno S, Sohrmann M *et al* (2003) Systematic functional analysis of the *Caenorhabditis elegans* genome using RNAi. *Nature* 421: 231–237

Keane OM, Toft C, Carretero-Paulet L, Jones GW, Fares MA (2014) Preservation of genetic and regulatory robustness in ancient gene duplicates of *Saccharomyces cerevisiae*. *Genome Res* 24: 1830–1841

Kuzmin E, Taylor JS, Boone C (2021) Retention of duplicated genes in evolution. *Trends Genet* 38: 883

Lawrence MS, Stojanov P, Mermel CH, Robinson JT, Garraway LA, Golub TR, Meyerson M, Gabriel SB, Lander ES, Getz G (2014) Discovery and saturation analysis of cancer genes across 21 tumour types. *Nature* 505: 495–501

Li Y, Roberts ND, Wala JA, Shapira O, Schumacher SE, Kumar K, Khurana E, Waszak S, Korbel JO, Haber JE *et al* (2020) Patterns of somatic structural variation in human cancer genomes. *Nature* 578: 112–121

López S, Lim EL, Horswell S, Haase K, Huebner A, Dietzen M, Mourikis TP, Watkins TBK, Rowan A, Dewhurst SM *et al* (2020) Interplay between whole-genome doubling and the accumulation of deleterious alterations in cancer evolution. *Nat Genet* 52: 283–293

Lord CJ, Quinn N, Ryan CJ (2020) Integrative analysis of large-scale loss-of-function screens identifies robust cancer-associated genetic interactions. *Elife* 9: e58925

Makino T, McLysaght A (2010) Ohnologs in the human genome are dosage balanced and frequently associated with disease. *Proc Natl Acad Sci USA* 107: 9270–9274

Martincorena I, Raine KM, Gerstung M, Dawson KJ, Haase K, Van Loo P, Davies H, Stratton MR, Campbell PJ (2017) Universal patterns of selection in cancer and somatic tissues. *Cell* 171: 1029–1041

McFarland CD, Mirny LA, Korolev KS (2014) Tug-of-war between driver and passenger mutations in cancer and other adaptive processes. *Proc Natl Acad Sci USA* 111: 15138–15143

McKinney W (2011) pandas: a foundational Python library for data analysis and statistics. *Python for High Performance and Scientific Computing*: 1–9

Meyers RM, Bryan JG, McFarland JM, Weir BA, Sizemore AE, Xu H, Dharia NV, Montgomery PG, Cowley GS, Pantel S *et al* (2017) Computational correction of copy number effect improves specificity of CRISPR–Cas9 essentiality screens in cancer cells. *Nat Genet* 49: 1779–1784

Muller FL, Colla S, Aquilanti E, Manzo VE, Genovese G, Lee J, Eisenson D, Narurkar R, Deng P, Nezi L *et al* (2012) Passenger deletions generate therapeutic vulnerabilities in cancer. *Nature* 488: 337–342

Oike T, Ogiwara H, Nakano T, Kohno T (2014) Proposal for a synthetic lethality therapy using the paralog dependence of cancer cells—response. *Cancer Res* 74: 4948–4949

O'Toole ÁN, Hurst LD, McLysaght A (2018) Faster evolving primate genes are more likely to duplicate. *Mol Biol Evol* 35: 107–118

Parrish PCR, Thomas JD, Gabel AM, Kamlapurkar S, Bradley RK, Berger AH (2021) Discovery of synthetic lethal and tumor suppressor paralog pairs in the human genome. *Cell Rep* 36: 109597

Pertesi M, Ekdahl L, Palm A, Johnsson E, Järvstråt L, Wihlborg A-K, Nilsson B (2019) Essential genes shape cancer genomes through linear limitation of homozygous deletions. *Commun Biol* 2: 262

Pujar S, O'Leary NA, Farrell CM, Loveland JE, Mudge JM, Wallin C, Girón CG, Diekhans M, Barnes I, Bennett R *et al* (2018) Consensus coding sequence (CCDS) database: a standardized set of human and mouse protein-coding regions supported by expert curation. *Nucleic Acids Res* 46: D221–D228

Ryan CJ, Mehta I, Kebabci N, Adams DJ (2023) Targeting synthetic lethal paralogs in cancer. *Trends Cancer Res* 9: 397–409

Seabold S, Perktold J (2010) Statsmodels: Econometric and statistical modeling with python. In *Proceedings of the 9th Python in Science Conference*, p 61. Scipy

Singh PP, Isambert H (2020) OHNOLOGS v2: a comprehensive resource for the genes retained from whole genome duplication in vertebrates. *Nucleic Acids Res* 48: D724–D730

Sondka Z, Bamford S, Cole CG, Ward SA, Dunham I, Forbes SA (2018) The COSMIC Cancer Gene Census: describing genetic dysfunction across all human cancers. *Nat Rev Cancer* 18: 696–705

 © 2023 The Authors

Thompson NA, Ranzani M, van der Weyden L, Iyer V, Offord V, Droop A, Behan F, Gonçalves E, Speak A, Iorio F *et al* (2021) Combinatorial CRISPR screen identifies fitness effects of gene paralogues. *Nat Commun* 12: 1302

Tian R, Abarientos A, Hong J, Hashemi SH, Yan R, Dräger N, Leng K, Nalls MA, Singleton AB, Xu K *et al* (2021) Genome-wide CRISPRi/a screens in human neurons link lysosomal failure to ferroptosis. *Nat Neurosci* 24: 1020–1034

Tilk S, Tkachenko S, Curtis C, Petrov DA, McFarland CD (2022) Most cancers carry a substantial deleterious load due to Hill-Robertson interference. *Elife* 11: e67790

Van den Eynden J, Basu S, Larsson E (2016) Somatic mutation patterns in hemizygous genomic regions unveil purifying selection during tumor evolution. *PLoS Genet* 12: e1006506

Van Loo P, Nordgard SH, Lingjærde OC, Russnes HG, Rye IH, Sun W, Weigman VJ, Marynen P, Zetterberg A, Naume B *et al* (2010) Allele-specific copy number analysis of tumors. *Proc Natl Acad Sci USA* 107: 16910–16915

VanderSluis B, Bellay J, Musso G, Costanzo M, Papp B, Vizeacoumar FJ, Baryshnikova A, Andrews B, Boone C, Myers CL (2010) Genetic interactions reveal the evolutionary trajectories of duplicate genes. *Mol Syst Biol* 6: 429

Vogelstein B, Papadopoulos N, Velculescu VE, Zhou S, Diaz LA Jr, Kinzler KW (2013) Cancer genome landscapes. *Science* 339: 1546–1558

Wang T, Birsoy K, Hughes NW, Krupczak KM, Post Y, Wei JJ, Lander ES, Sabatini DM (2015) Identification and characterization of essential genes in the human genome. *Science* 350: 1096–1101

Wang Y, Jiang B, Wu Y, He X, Liu L (2022) Rapid intraspecies evolution of fitness effects of yeast genes. *Genome Biol Evol* 14: evac061

Weghorn D, Sunyaev S (2017) Bayesian inference of negative and positive selection in human cancers. *Nat Genet* 49: 1785–1788

White JK, Gerdin A-K, Karp NA, Ryder E, Buljan M, Bussell JN, Salisbury J, Clare S, Ingham NJ, Podrini C *et al* (2013) Genome-wide generation and systematic phenotyping of knockout mice reveals new roles for many genes. *Cell* 154: 452–464

Wolfe KH, Shields DC (1997) Molecular evidence for an ancient duplication of the entire yeast genome. *Nature* 387: 708–713

Zack TI, Schumacher SE, Carter SL, Cherniack AD, Saksena G, Tabak B, Lawrence MS, Zhsng C-Z, Wala J, Mermel CH *et al* (2013) Pan-cancer patterns of somatic copy number alteration. *Nat Genet* 45: 1134–1140

Zapata L, Pich O, Serrano L, Kondrashov FA, Ossowski S, Schaefer MH (2018) Negative selection in tumor genome evolution acts on essential cellular functions and the immunopeptidome. *Genome Biol* 19: 67

Zerbino DR, Achuthan P, Akanni W, Amode MR, Barrell D, Bhai J, Billis K, Cummins C, Gall A, Girón CG *et al* (2018) Ensembl 2018. *Nucleic Acids Res* 46: D754–D761

Zhang J (2003) Evolution by gene duplication: an update. *Trends Ecol Evol* 18: 292–298

License: This is an open access article under the terms of the Creative Commons Attribution License, which permits use, distribution and reproduction in any medium, provided the original work is properly cited.

Expanded View Figures

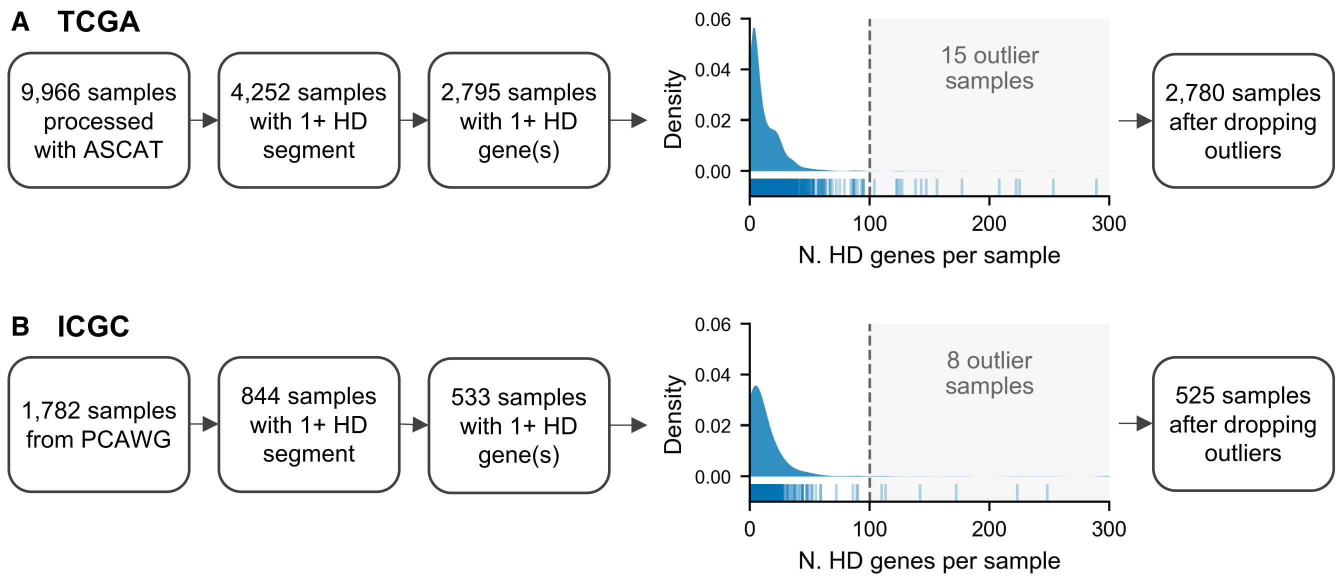

Figure EV1. Workflow for tumor samples used in this study.

A Flowchart for the number of TCGA tumor samples used for HD analysis. The density/tick plot shows the distribution of the number of HD genes per sample; only samples with at least 1 gene HD are shown in this plot. Samples to the right of the dotted line were marked as outliers and dropped from further analysis.

B Same as (A) but for ICGC tumor samples from the PCAWG study.

Figure EV2. Gene-level homozygous deletion frequency across the genome.

Line plots showing, for each gene plotted according to its genomic location, the number of TCGA tumor samples in which the gene is fully homozygously deleted. The number of samples is capped at 40 for visualization purposes. Orange ticks show the location of all TSGs with at least three HDs; four TSG peaks are annotated with the gene symbol and number of HDs. Fragile sites are denoted by blue ticks and centromeres by dotted gray lines.

© 2023 The Authors

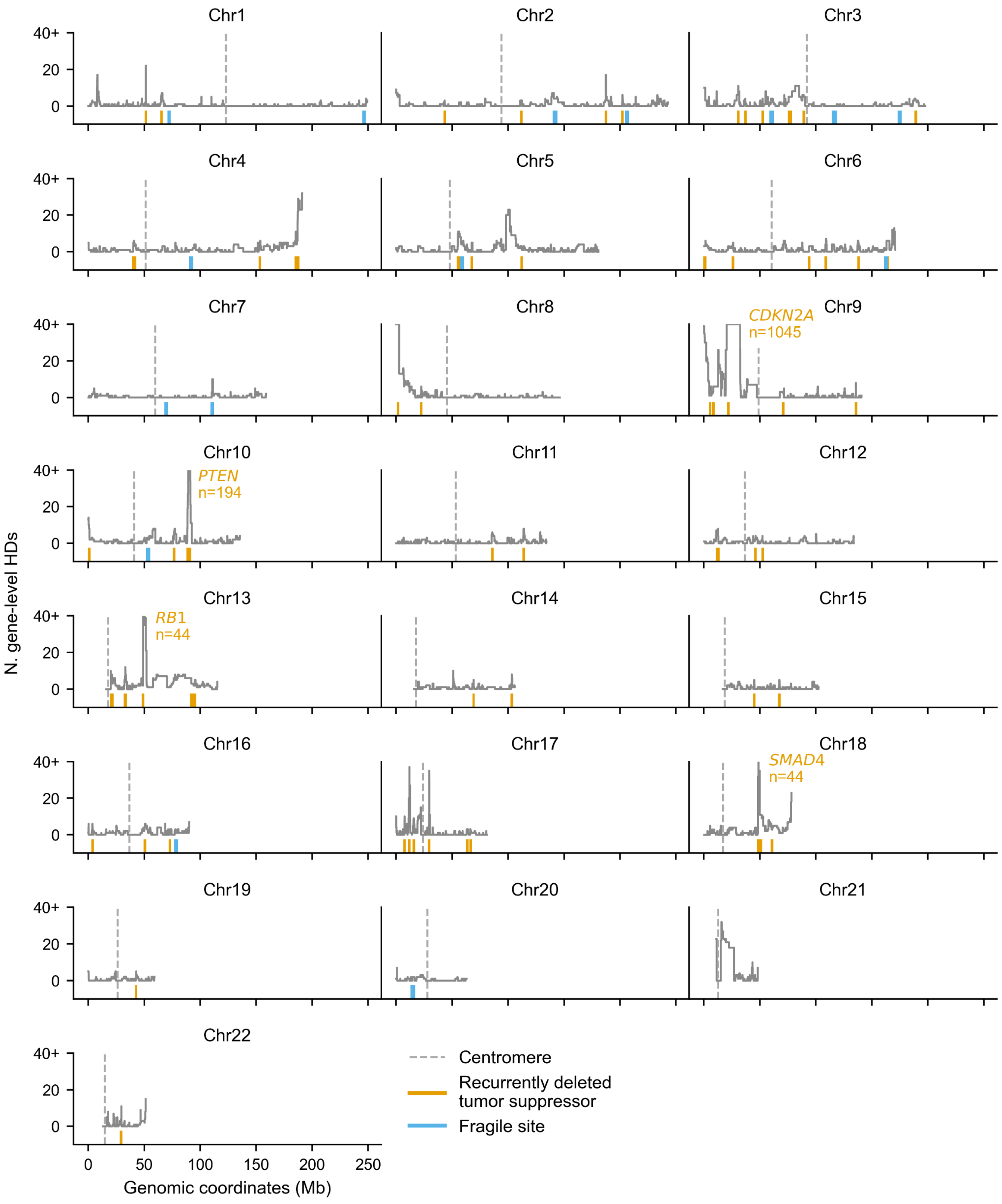
Chr1
Chr2
Chr3
Chr4
Chr5
Chr6
Chr7
Chr8
Chr9
CDKN2A
n=1045
Chr10
PTEN
n=194
Chr11
Chr12
Chr13
RB1
n=44
Chr14
Chr15
Chr16
Chr17
Chr18
SMAD4
n=44
Chr19
Chr20
Chr21
Chr22
40+
20
0
N. gene-level HDs
0
50
100
150
200
250
Genomic coordinates (Mb)
Centromere
Recurrently deleted tumor suppressor
Fragile site

Figure EV2.

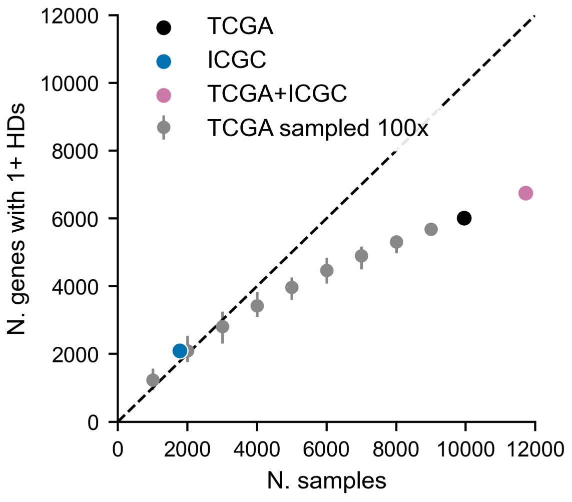

Figure EV3. Gene HD saturation analysis.

Dot plot showing the number of unique gene HDs observed (y-axis) for increasing numbers of tumor samples (x-axis). The black dot shows the actual number of unique gene HDs observed in the TCGA cohort. Gray dots are the result of down-sampling the TCGA cohort and error bars indicate the minimum and maximum values observed from 100 random samplings. The blue dot shows the actual number of unique gene HDs observed in the ICGC cohort, while the pink dot indicates the number of gene HDs that are observed when combining the TCGA and ICGC cohorts into one dataset.

Figure EV4. Paralog passengers are more likely to be subject to homozygous but not hemizygous deletion.

A Similar to Fig 2A but for TCGA tumor samples stratified by cancer type. Top: Percentage of passenger (non-driver) genes that are deleted in either zero (0) or at least one (1+) TCGA samples. The number of TCGA tumor samples for each cancer type considered is shown. Bottom: For passenger genes grouped according to never deleted (0) or deleted at least once (1+), the solid blue line shows the percentage of genes in each group that are paralogs. The dashed line shows the percentage of all passenger genes that are paralogs. Annotations show the Odds Ratio (OR) for a Fisher's Exact Test comparing the percentage of paralogs among genes with 0 vs. 1+ HDs; asterisk (*) indicates $P < 0.05$.

B Boxplots showing the number of TCGA samples in which gene-level LOH is observed for singleton vs. paralog passenger genes. LOH here is identified when one allele has copy number equal to 0. From left to right the box plots show all LOH segments, only focal LOH segments, copy loss segments (total copy number = 1), and copy neutral LOH segments (total copy number = 2). The boxes represent the first and third quartiles (Q1 and Q3) of the distribution, the horizontal black line the median, and the whiskers extend up to 1.5*the interquartile range past Q1 and Q3. Outliers are shown as gray circles. The P-values shown are from MWU tests comparing paralogs and singletons.

C Same as (B) but for ICGC tumor samples.

 © 2023 The Authors

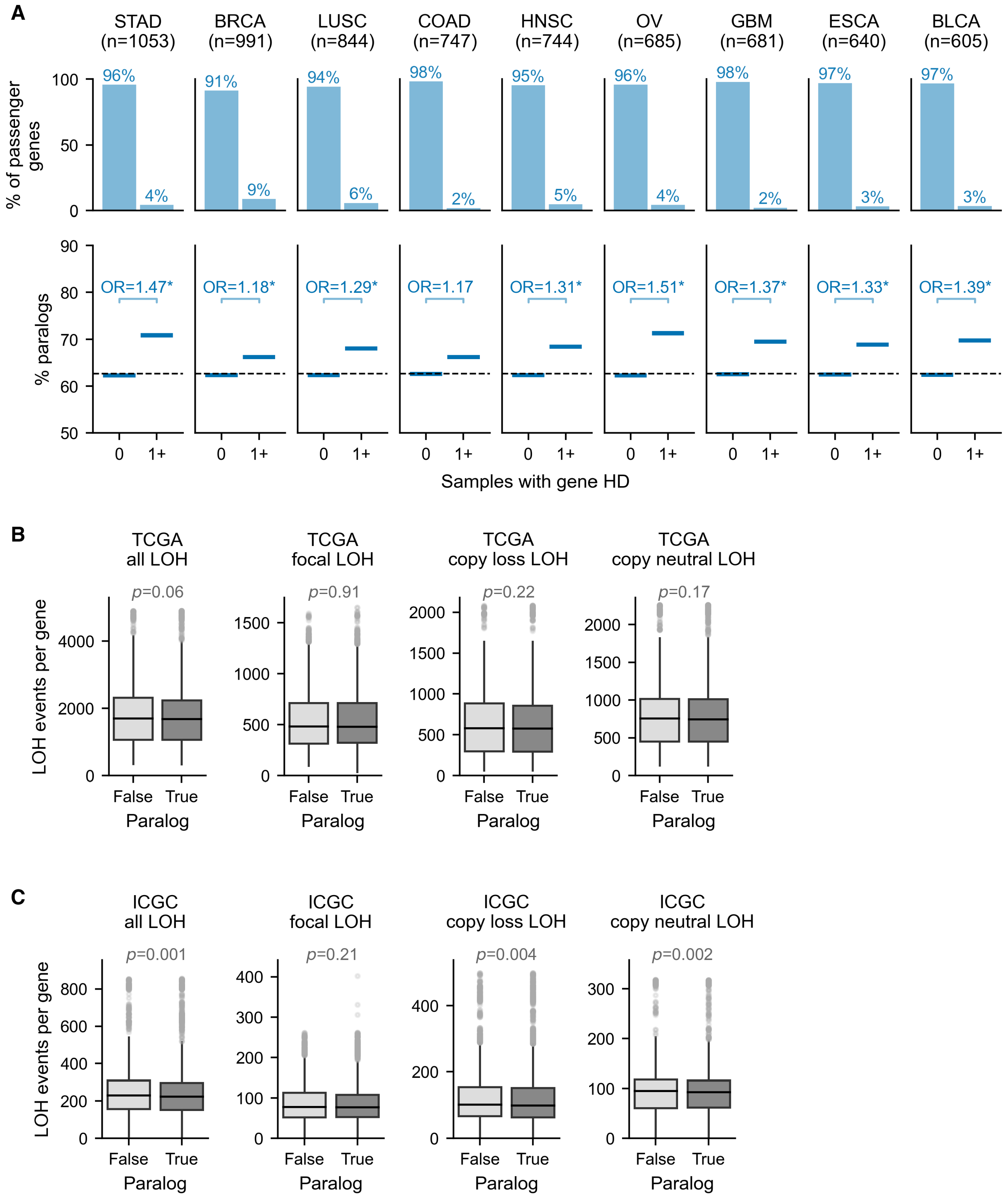
A
STAD (n=1053)
BRCA (n=991)
LUSC (n=844)
COAD (n=747)
HNSC (n=744)
OV (n=685)
GBM (n=681)
ESCA (n=640)
BLCA (n=605)
% of passenger genes
96% 4%
91% 9%
94% 6%
98% 2%
95% 5%
96% 4%
98% 2%
97% 3%
97% 3%
% paralogs
OR=1.47*
OR=1.18*
OR=1.29*
OR=1.17
OR=1.31*
OR=1.51*
OR=1.37*
OR=1.33*
OR=1.39*
Samples with gene HD
B
TCGA all LOH
TCGA focal LOH
TCGA copy loss LOH
TCGA copy neutral LOH
p=0.06
p=0.91
p=0.22
p=0.17
LOH events per gene
Paralog
False True
C
ICGC all LOH
ICGC focal LOH
ICGC copy loss LOH
ICGC copy neutral LOH
p=0.001
p=0.21
p=0.004
p=0.002
LOH events per gene
Paralog
False True

Figure EV4.

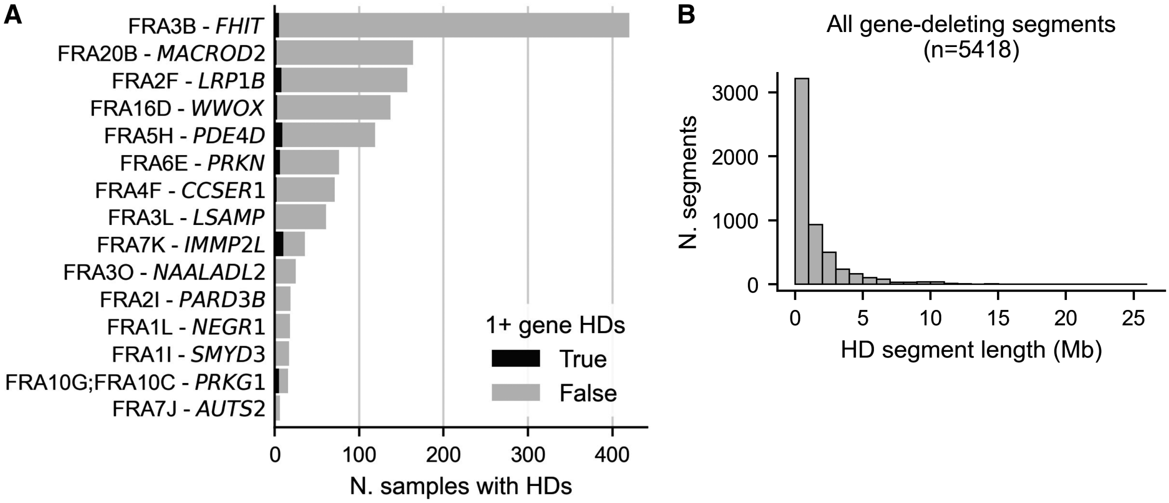

Figure EV5. Characterization of HD segments.

A Bar plot showing the number of TCGA samples with an HD overlapping each of 15 major fragile sites, with the section of the bar colored black indicating the number of HDs that result in at least 1 full gene HD. Fragile sites are listed with their name (e.g. FRA3B) and the longest gene they contain (e.g. *FHIT*).

B Histogram showing the distribution of the lengths of all HD segments from both cohorts that fully delete at least 1 gene.

 © 2023 The Authors
