# Supplementary material for: Paralog dispensability shapes homozygous deletion patterns in tumor genomes
Source: Mol Syst Biol. 2023 Nov 14;19(12):e11987. doi: 10.15252/msb.202311987 (PMC10698506; doi:10.15252/msb.202311987)
Supplement: Supplementary file 6 [file MSB-19-e11987-s003.pdf]

Paralog dispensability shapes homozygous deletion patterns in tumor genomes

Barbara De Kegel and Colm Ryan
DOI: 10.15252/msb.202311987

Corresponding author(s): Colm Ryan (colm.ryan@ucd.ie)

Review Timeline:

Transfer from Review Commons:	5th Sep 23
Editorial Decision:	16th Oct 23
Revision Received:	24th Oct 23
Accepted:	26th Oct 23

Editor: Maria Polychronidou

Transaction Report: This manuscript was transferred to Molecular Systems Biology following peer review at Review Commons.

Review #1

1. Evidence, reproducibility and clarity:

Evidence, reproducibility and clarity (Required)

Summary:

In this study, the authors delineate the association of paralog dispensability with the frequency of homozygous deletions (HDs) and thereby show that paralog dispensability can play a significant role in shaping tumor genomes. The authors analyzed the strength of negative selection on the paralogs relative to the singletons using frequencies of the homozygous deletions (HD). The study focused on HDs because they ensure a complete loss of function, unlike other mutational aberrations that can be masked because of haplo-sufficiency. While accounting for potential confounding factors, authors find that paralogs tend to have a relatively high frequency of HDs, suggesting a relaxed negative selection. Furthermore, the authors specifically attribute this association to the dispensable paralogs by analyzing gene inactivation data generated from multiple experimental systems. Overall, the findings of this study can potentially have significant implications in cancer biology field and specifically to the researchers studying cancer genome evolution.

Major comments:

1. To dissect further which dispensable paralogs are more likely to be associated with a high HD frequency, synthetic lethal paralogs could be compared with non-synthetic lethal ones.
In the section titled 'Homozygous deletion frequency of paralog passengers is influenced by paralog properties' (begins from line #289), authors have shown that paralogs with a high frequency of HDs are more likely to have the properties of dispensability (in Figure 4). It seems that all of those properties are also associated with synthetic lethality as the authors identified in their previous study (DeKegel et al. 2021). Furthermore, as shown in the subsequent section ('Essential paralogs are less frequently homozygously deleted than non-essential paralogs', begins from line #344), the high HD is associated with the dispensable paralogs. Some of those dispensable paralogs are expected to be synthetic lethal. Therefore, the association of paralogs with a high frequency of HDs with experimentally validated or predicted sets of synthetic lethal paralogs could be tested. This may help authors to contextualize their findings in terms of genetic interactions between paralogs.

Minor comments:

1. The number of TCGA and ICGC tumor samples analyzed:
As mentioned in the Results section (line #106), 9966 tumor samples were analyzed. However, the sample size mentioned in Figure 2A is 9951. Is the lower number shown in the figure due to the filtering procedure mentioned in the Methods section (line #455)? The change in sample sizes could be explained. A similar difference in sample sizes exists for the ICGC data also.
2. The rationale behind setting the threshold at 100 HD genes to classify 'hyper-deleted' samples for TCGA (line #462) and ICGC data (line #473) could be explained.
3. Citation for DepMap is missing (caption of Figure 5).

Referees cross-commenting

Along the lines of Reviewer #3's second major comment, I have a suggestion, the possible benefits of which would depend on the target audience to which the authors intend to communicate their study.

I would suggest including a brief comparison of the findings of this study which deal with human paralogs, with the findings in model organisms such as yeast, perhaps in the discussion section. To facilitate such a comparison, authors could try measuring the enrichments of, for example, molecular functions, gene families, types of genetic interactions, etc., among the paralogs associated with a high frequency of HDs and then discussing their comparison with what is known in the literature for paralogs in other model organisms that tend to be frequently deleted.

Such a comparison could be of interest to the community of researchers working on other model organisms and put this study in a much broader context. However, as I said before, this would depend on the authors' intended target audience.

2. Significance:

Significance (Required)

As the authors note in their manuscript, it is expected that paralog dispensability could be associated with the relaxed negative selection in tumor genomes because (1) paralogs are prevalent in the human genome, and (2) many of them are dispensable, as apparent from the large-scale gene inactivation screens in hundreds of cancer cell lines (Blomen et al. 2015, Wang et al. 2015, Dandage and Landry 2019, De Kegel and Ryan 2019). However, direct mapping of this association, while importantly accounting for potential confounding factors, has been lacking.

As a researcher with prior experience in the research topics such as gene duplication and genetic interactions, it appears to me that this study presents formal proof of the important association between paralog dispensability and tumor genome evolution which could be of major implication for the research community of cancer biology field and specifically to the researchers dealing with the topics such as cancer evolution, copy number alterations in cancer genomes, and synthetic lethality-based precision oncology therapeutics.

3. How much time do you estimate the authors will need to complete the suggested revisions:

Estimated time to Complete Revisions (Required)

(Decision Recommendation)

Less than 1 month

4. *Review Commons* values the work of reviewers and encourages them to get credit for their work. Select 'Yes' below to register your reviewing activity at Web of Science Reviewer Recognition Service (formerly Publons); note that the content of your review will not be visible on Web of Science.

Web of Science Reviewer Recognition

No

Review #2

1. Evidence, reproducibility and clarity:

Evidence, reproducibility and clarity (Required)

Summary

Here, De Kegel & Ryan analyse thousands of tumour samples from the TCGA and

ICGC projects to identify homozygously deleted genes, finding that about 40% of protein-coding genes are deleted in at least one sample. They find homozygously deleted genes to be enriched for paralogous genes, and further, more frequently deleted genes are increasingly likely to be paralogs. The authors then test the influence of several factors on the likelihood of being deleted, including gene length, distance to a fragile site or chromosomal region, and distance to a recurrently deleted tumour suppressor gene (TSG). They find that proximity of a TSG, telomere, centromere, and fragile site all increase likelihood of being deleted in a sample, as does gene length. Having a paralog also remains an important predictor of deletion after accounting for these other factors. Additionally, the more similar in sequence the closest paralog is to the gene and having a larger gene family size are also predictive of deletion. Conversely, if a gene is a whole genome duplicate as opposed to a small-scale duplicate, it is less likely to be deleted. Finally, the authors test the hypothesis that paralogs that are deleted in cancer are less likely to be essential and find that this is indeed the case.

Comments

The authors have done a good job of identifying trends of paralog deletion in cancer samples and the factors influencing them. The results are well described and presented and support the conclusions. I like the inclusion of the saturation analysis as an estimate of what to expect given current and potential future sample sizes, and I appreciate the inclusion of a WGD/SSD paralog distinction. The data and methods are sufficiently detailed. I have a few minor comments below.

1. Around line 160 in the text and supplemental figure 4A, the authors test if the trends they see are observed across individual cancer types. With 9 of 33 cancer types reaching a sample size threshold, 8 of 9 comparisons are significant. The authors do not state correcting for multiple testing.
2. I initially misunderstood the hemizygously deletion analysis, thinking the analysis in supplement figure 4B/C was asking if a sample has any singleton or any paralog deleted and comparing the number of samples with any deletion of either - given the number of genes deleted per sample this wouldn't make sense as a good test. I think the authors are actually comparing the number of loss-of-hemizygosity events per gene and grouping by paralog/singleton. I think this is a good analysis, but I think it would be helpful to clarify this in the text and figure legend e.g. "Samples w/ gene LOH" could be "LOH events per gene" or something similar.
3. Occasionally, I wanted some more detail in the text for context, which was sometimes later provided - e.g. I noted when reading about line 125 that I was curious

at this point how often TSGs occurred on segments, and this was later provided on line 241. Similarly, around line 114 I was curious how many genes are typically deleted per HD segment, for which the median value was provided on line 206 (and distribution in supplemental figure 1), and again for hemizygous deletions. I think sometimes it would be helpful to provide this context earlier in the text to aid interpretation of the results.
4. In the discussion, on line 420, the authors include the point that a paralog might not be required at all in a tumour cell and therefore easily deleted. I think this possibility could be expanded on here and in the introduction/results section, as it is an important point. I think it would be helpful to include more about the possibility that a paralog might be deleted in a tumour cell because it is simply not required or that is more likely to have less of a phenotypic impact compared to a singleton, and that this could be a reason for the observed enrichment of paralogs in deleted genes. A citation to support this point could be Áine N O'Toole, Laurence D Hurst, Aoife McLysaght, Faster Evolving Primate Genes Are More Likely to Duplicate, Molecular Biology and Evolution, Volume 35, Issue 1, January 2018, Pages 107-118, https://doi.org/10.1093/molbev/msx270. Duplicate genes can be duplicates because copy number variation of them has minimal impact.

Referees cross-commenting

I agree, that's a helpful suggestion from reviewer 1.

Reviewer 3's suggestion regarding age of the two whole genome duplication events is quite difficult to unpick as the duplication events seem to have happened relatively close in time to each other while rediploidisation of the first was occurring. Additionally, paralogs from SSDs tend to be more similar in sequence simply because the two WGD events are relatively old while SSDs can occur at any time up to present. They're therefore biased by young duplicates that have not had the opportunity to diverged much and decrease in sequence similarity.

2. Significance:

Significance (Required)

This is a novel study as it examines the frequency of paralog deletion in cancer samples and the factors influencing it, building upon work already conducted in cancer cell lines. This study extends the knowledge of the field confirming previous trends observed in cell lines, this time in actual cancer samples. It confirms that paralogs are more dispensable than singletons, likely because they have a similar

counterpart that can provide some level of functional redundancy. The more similar the closest paralog, the more likely it is to be deleted provides support for this.

It is certainly limited by the number of samples currently available in the two cancer sample projects included but the authors attempt to quantify how limiting this sample size is by conducting a saturation analysis using down-sampling to estimate how many gene deletions one can expect from different numbers of samples. This is important as the lack of observance of many gene deletions is likely due to the limited sample size and not due to negative selection. This low observance of gene deletions disappointingly limits further testing beyond single paralogs to consider the deletion effects of multiple gene family members and more directly test evidence of functional redundancy between paralogs. The authors provide a good discussion of the limitations of their study.

The results are of interest to evolutionary biologists and cancer biologists. Those with an interest in duplicate genes, and/or factors affecting gene loss in tumours will be interested in this work.

My field of expertise is molecular evolution, gene duplication and copy number variation.

3. How much time do you estimate the authors will need to complete the suggested revisions:

Estimated time to Complete Revisions (Required)

(Decision Recommendation)

Less than 1 month

4. *Review Commons* values the work of reviewers and encourages them to get credit for their work. Select 'Yes' below to register your reviewing activity at Web of Science Reviewer Recognition Service (formerly Publons); note that the content of your review will not be visible on Web of Science.

Web of Science Reviewer Recognition

Yes

Review #3

1. Evidence, reproducibility and clarity:

Evidence, reproducibility and clarity (Required)

Thank you review "Paralog dispensability shapes homozygous deletion patterns in tumor genomes" by DeKegel et al. This manuscript uses TCGA and ICGC tumor data to show evidence for paralog dispensability. They analyze the rate of homozygous deletions and show that it is higher for paralogs compared to singletons. Their findings are robust to a number of confounding variables that they take into account e.g. distance to tumor suppressor, telomere, centromere or fragile site. They show that paralogs that belong to large families and have higher sequence identity tend to show more dispensability and these dispensable paralogs are less likely to be WGD.

Major comments.

1. Does the finding pertaining to lack of enrichment of paralogs in regions LOH take into account whether LOH is copy neutral or not i.e. how does dosage affects this finding? Is it possible that there is a difference in paralog rate in LOH that results in total copy 1 and that the presence of copy neutral LOH masks the effect? Also, Integration of gene expression dataset would be helpful to resolve the difference between dosage paralog that compensate of the lack of their sister by upregulating their gene expression.
2. Is the finding that paralogs are depleted among WGD is influenced by the age of WGD since there are 2 WGD events? Do SSD tend to be more or less similar by seq than WGD? This should be explored further since this observation is the opposite of what is observed in model organisms such as yeast whereby SSD are less functionally similarthan WGD and often show properties similar to singletons than WGD.

Minor comments

1. There is a missing reference on line 55.

Referees cross-commenting

That's a good suggestion by reviewer 1. Homozygous deletion collection is available in yeast so these data can be used directly in addition tot he haploid gene deletion collection data.

Since authors of this manuscript included in their analysis the comparison of WGD and SSD then they should do it more thoroughly. It is not sufficient what they presented here especially given that it contradicts the findings from model organisms.

2. Significance:

Significance (Required)

This work provides the first systematic assessment of paralog dispensability specifically looking at homozygous deletions of paralogs across primary tumor samples and builds on the existing findings in cancer cell lines. It will be broadly interesting to those studying duplicated gene evolution and genome robustness. My expertise is in complex genetic networks in yeast and human cancer as well as genome evolution.

3. How much time do you estimate the authors will need to complete the suggested revisions:

Estimated time to Complete Revisions (Required)

(Decision Recommendation)

Less than 1 month

4. *Review Commons* values the work of reviewers and encourages them to get credit for their work. Select 'Yes' below to register your reviewing activity at Web of Science Reviewer Recognition Service (formerly Publons); note that the content of your review will not be visible on Web of Science.

Web of Science Reviewer Recognition

Yes

Reviewer #1 (Evidence, reproducibility and clarity (Required)):

Summary:
In this study, the authors delineate the association of paralog dispensability with the frequency of homozygous deletions (HDs) and thereby show that paralog dispensability can play a significant role in shaping tumor genomes. The authors analyzed the strength of negative selection on the paralogs relative to the singletons using frequencies of the homozygous deletions (HD). The study focused on HDs because they ensure a complete loss of function, unlike other mutational aberrations that can be masked because of haplo-sufficiency. While accounting for potential confounding factors, authors find that paralogs tend to have a relatively high frequency of HDs, suggesting a relaxed negative selection. Furthermore, the authors specifically attribute this association to the dispensable paralogs by analyzing gene inactivation data generated from multiple experimental systems. Overall, the findings of this study can potentially have significant implications in cancer biology field and specifically to the researchers studying cancer genome evolution.

We thank the reviewer for the careful reading and positive assessment of our manuscript

Major comments:

1. To dissect further which dispensable paralogs are more likely to be associated with a high HD frequency, synthetic lethal paralogs could be compared with non-synthetic lethal ones.

In the section titled 'Homozygous deletion frequency of paralog passengers is influenced by paralog properties' (begins from line #289), authors have shown that paralogs with a high frequency of HDs are more likely to have the properties of dispensability (in Figure 4). It seems that all of those properties are also associated with synthetic lethality as the authors identified in their previous study (DeKegel et al. 2021). Furthermore, as shown in the subsequent section ('Essential paralogs are less frequently homozygously deleted than non-essential paralogs', begins from line #344), the high HD is associated with the dispensable paralogs. Some of those dispensable paralogs are expected to be synthetic lethal. Therefore, the association of paralogs with a high frequency of HDs with experimentally validated or predicted sets of synthetic lethal paralogs could be tested. This may help authors to contextualize their findings in terms of genetic interactions between paralogs.

We thank the reviewer for highlighting the potential relationship with our previous work. We agree that many of these properties are associated with synthetic lethality, but we note that they are also associated with single gene essentiality. This makes the relationship between synthetic lethality, essentiality, and deletion frequency somewhat difficult to dissect.

Nonetheless we have tested, in a number of ways, whether there is a relationship between a paralog having a reported/predicted synthetic lethality and being homozygously deleted. We find no obvious connection between the two.

We first tested using a set of synthetic lethal interactions identified by integrating molecular profiling data with genome wide CRISPR screens in a large panel of cancer cell lines (the data used to train the classifier in De Kegel et al, 2021). As there is an ascertainment bias in this dataset (paralogs must have frequent loss of function alterations / silencing to be tested) we restrict our analysis to only those paralog pairs tested for synthetic lethality. We identified no clear pattern:

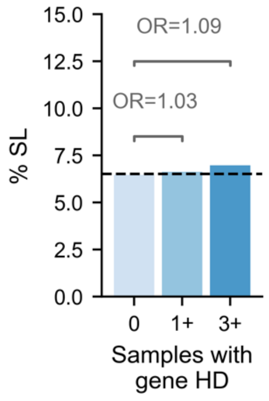

On the y-axis is the percentage of genes in each bin that have a synthetic lethal paralog. ‘OR’ indicates the odds ratio from a Fisher’s exact test. We note that the p-value from this test, and all of the tests used to determine enrichment of synthetic lethality shown below, is not significant (p>0.05).

We next tested using an integrated set of four combinatorial CRISPR screens (aggregated in De Kegel et al) where we considered a pair to be synthetic lethal if it was a hit in any screen and not synthetic lethal if it was screened at least once and never identified as a hit. Again we restrict our analysis to paralogs that were present in this dataset to prevent issues with ascertainment bias:

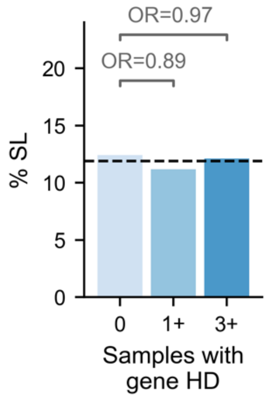

We further tested using a consensus dataset derived from the same combinatorial screens, where a pair were marked as synthetic lethal if they were identified as a hit in at least two screens and not synthetic lethal if they were screened at least twice and never identified as a hit. Again we restrict our analysis to paralogs that were present in this dataset:

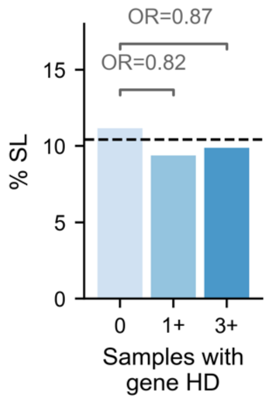

We finally tested using our predicted synthetic lethal interactions – annotating the top 3% of predictions as synthetic lethal and the remainder as non-synthetic lethal. The 3% threshold is similar to the observed frequency of synthetic lethality in the training set. In this case, as this dataset covers all paralogs considered, no restriction was necessary:

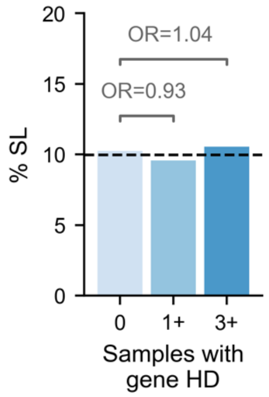

None of the above analyses revealed a clear relationship between deletion frequency and synthetic lethality. A caveat of these analyses is that none of the experimental datasets are complete (covering only a minority of all paralog pairs) and they are all somewhat noisy. Furthermore, as we show in our modelling analysis (Fig S3) the observed homozygous deletions are far from saturating.

However we think there may be a simpler explanation, beyond limitations of the data, for why we do not observe a relationship between HDs and synthetic lethality.

As the reviewer notes, there is evidence in cell lines that one reason paralogs are more dispensable than singletons is because of buffering / redundant relationships as revealed by synthetic lethal interactions. These relationships therefore provide an explanation for why some paralogs are dispensable. As our primary claim is that paralogs are more frequently deleted because they are more dispensable we might anticipate a relationship between deletion frequency and synthetic lethality. However, by definition, synthetic lethal interactions can only be observed for non-essential (dispensable) genes. Therefore when analysing the overlap with synthetic lethal interactions we are primarily restricting our analyses to genes that are already individually dispensable. Consequently we might not anticipate observing any enrichment. The buffering relationship revealed by synthetic lethality provides an

explanation for why a paralog is dispensable but once we are restricting our analysis to dispensable paralogs we do not necessarily expect to see further enrichment.

We think that an ideal way to explore this question further would be to look at selection on deletions of pairs of paralogs – we anticipate that if a gene is dispensable because of paralog buffering then both paralogs should not be deleted simultaneously. However, the current copy number datasets are too small to evaluate such pairwise relationships. This is discussed in manuscript as follows:

Analyzing the frequency with which two members of a paralog family are lost would provide more direct insight into the contribution of paralog redundancy, but due to the overall rarity of passenger gene HDs, we cannot make a comprehensive assessment of co-deletions here – e.g. among paralog pairs where both genes are non-drivers, and not on the same chromosome, only two pairs are co-deleted in at least one TCGA sample. Larger cohorts would also allow us to search for patterns of mutual exclusivity of HDs to identify genetic interactions – this has been applied for identifying interactions between driver genes [57,58], but is more challenging for interactions between non-driver genes, which are much less frequently altered.

Minor comments:
1. The number of TCGA and ICGC tumor samples analyzed:
As mentioned in the Results section (line #106), 9966 tumor samples were analyzed. However, the sample size mentioned in Figure 2A is 9951. Is the lower number shown in the figure due to the filtering procedure mentioned in the Methods section (line #455)? The change in sample sizes could be explained. A similar difference in sample sizes exists for the ICGC data also.

The difference was indeed due to filtering process, but numbers were only provided in the methods. We have now addressed this in the main text :

After removing a small number of 'hyper-deleted' samples (see Methods) we retained 9,951 samples for further analysis.

2. The rationale behind setting the threshold at 100 HD genes to classify 'hyper-deleted' samples for TCGA (line #462) and ICGC data (line #473) could be explained.

We excluded hyper-deleted samples to avoid any individual sample having undue influence on the genes observed to be ever deleted or indeed to influence the overall patterns observed. It is also common in analyses of selection in tumours that make use of mutational profiles (rather than copy number profiles) to exclude hypermutated samples (e.g. Martincorena et al, Cell 2017; Lopez et al, Nature 2020). However the exact threshold of 100 samples was somewhat arbitrary and this query prompted us to assess whether it had any significant impact on the results.

We therefore repeated all analyses using a more stringent threshold (50 samples) and also without thresholding. Although the exact percentages and odds-ratios vary somewhat with the different thresholds, all major conclusions are still supported. Below we include all major figures redrawn using multiple thresholds.

We appreciate that this was minor comment and that reviewer did not request this new analysis, but in the absence of a strong justification for a single threshold we felt it appropriate to assess multiple thresholds (and none).

Figure 2

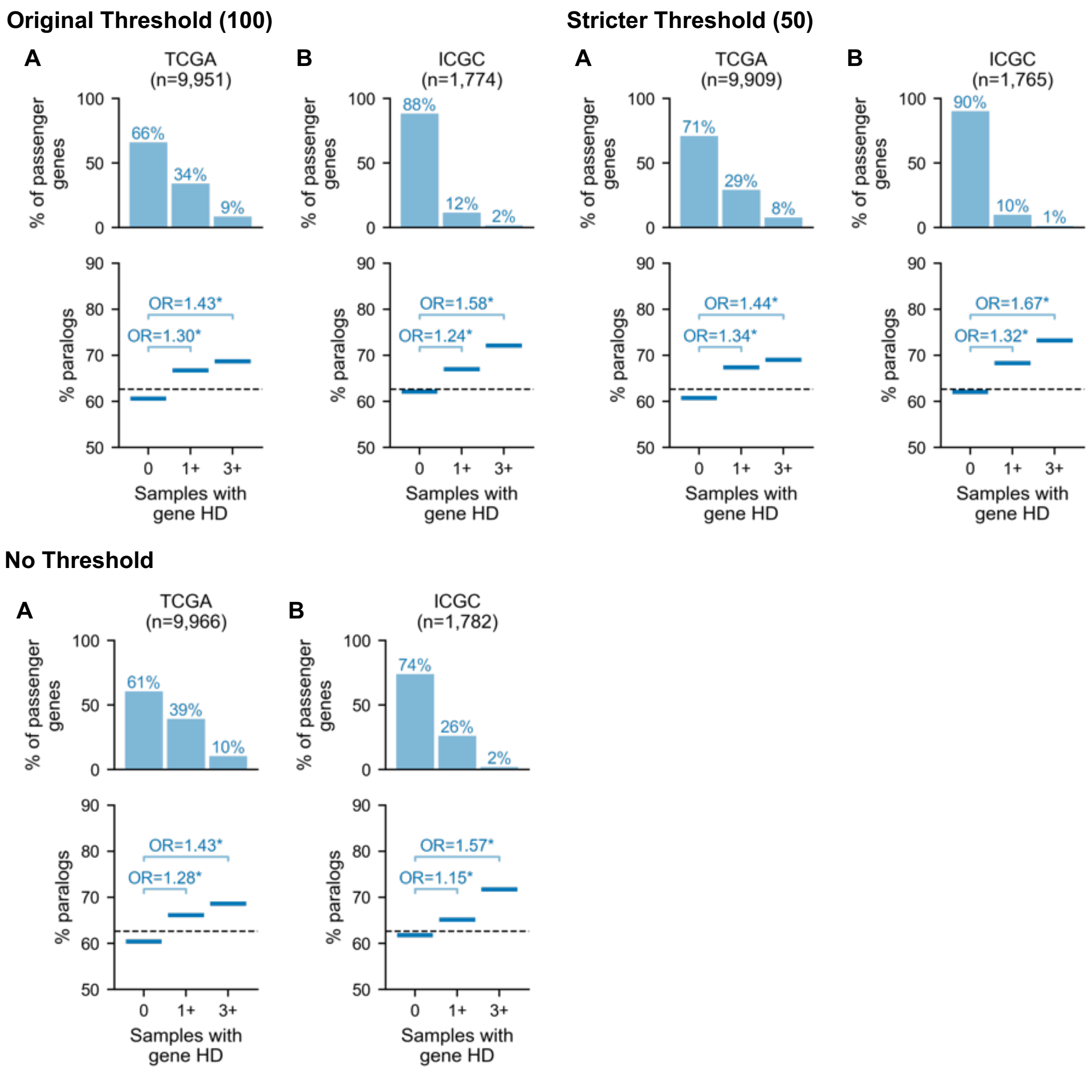

Figure 3C

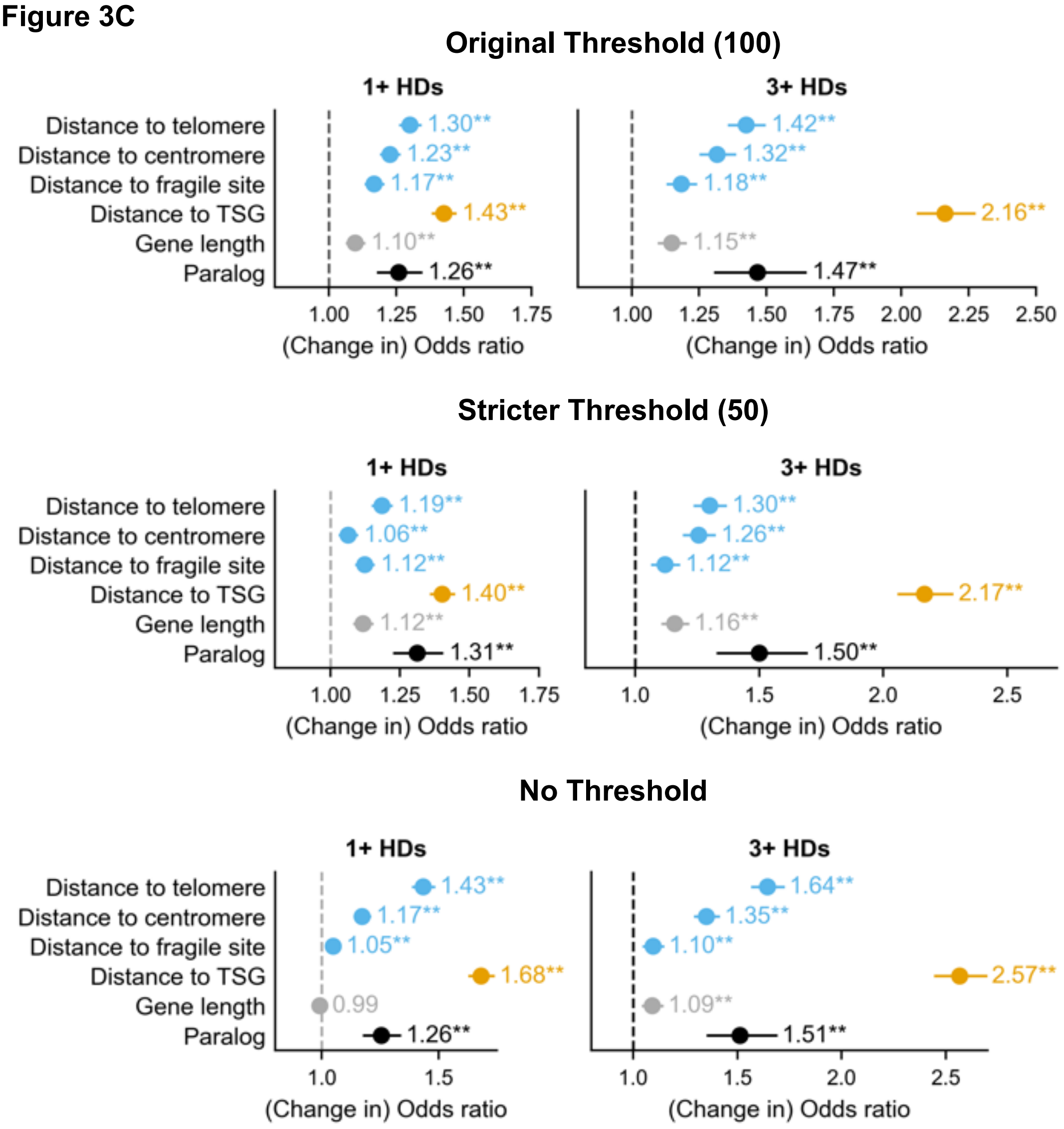

Original Threshold (100)
1+ HDs
3+ HDs
Distance to telomere
Distance to centromere
Distance to fragile site
Distance to TSG
Gene length
Paralog
1.30**
1.23**
1.17**
1.43**
1.10**
1.26**
1.42**
1.32**
1.18**
2.16**
1.15**
1.47**
1.00
1.25
1.50
1.75
2.00
2.25
2.50
(Change in) Odds ratio
Stricter Threshold (50)
1+ HDs
3+ HDs
Distance to telomere
Distance to centromere
Distance to fragile site
Distance to TSG
Gene length
Paralog
1.19**
1.06**
1.12**
1.40**
1.12**
1.31**
1.30**
1.26**
1.12**
2.17**
1.16**
1.50**
1.00
1.25
1.50
1.75
1.0
1.5
2.0
2.5
(Change in) Odds ratio
No Threshold
1+ HDs
3+ HDs
Distance to telomere
Distance to centromere
Distance to fragile site
Distance to TSG
Gene length
Paralog
1.43**
1.17**
1.05**
1.68**
0.99
1.26**
1.64**
1.35**
1.10**
2.57**
1.09**
1.51**
1.0
1.5
2.0
2.5
(Change in) Odds ratio

Figure 4 A-C

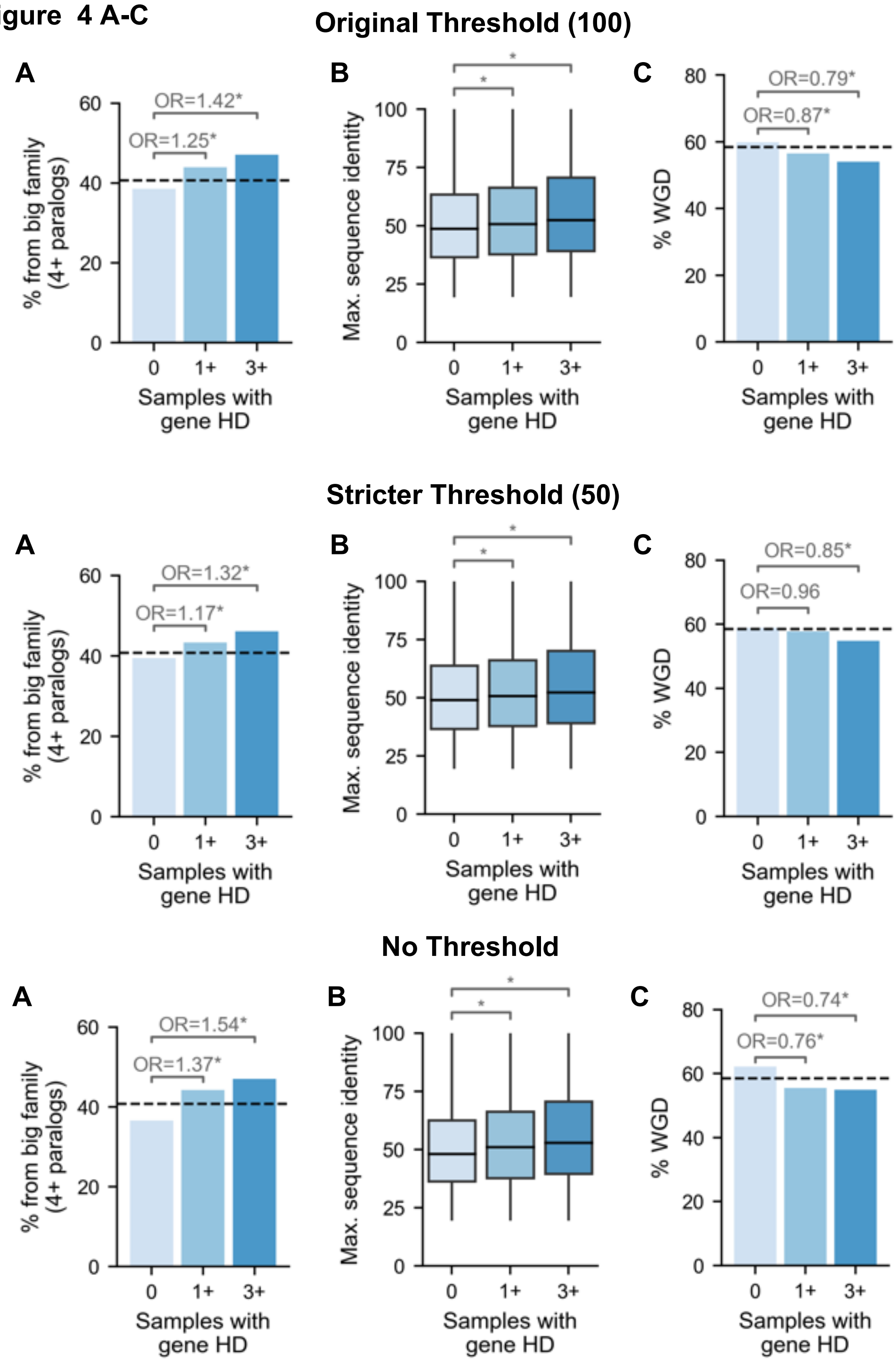
Original Threshold (100)
A
OR=1.42*
OR=1.25*
% from big family (4+ paralogs)
60
40
20
0
0
1+
3+
Samples with gene HD
B
*
*
Max. sequence identity
100
75
50
25
0
0
1+
3+
Samples with gene HD
C
OR=0.79*
OR=0.87*
% WGD
80
60
40
20
0
0
1+
3+
Samples with gene HD
Stricter Threshold (50)
A
OR=1.32*
OR=1.17*
% from big family (4+ paralogs)
60
40
20
0
0
1+
3+
Samples with gene HD
B
*
*
Max. sequence identity
100
75
50
25
0
0
1+
3+
Samples with gene HD
C
OR=0.85*
OR=0.96
% WGD
80
60
40
20
0
0
1+
3+
Samples with gene HD
No Threshold
A
OR=1.54*
OR=1.37*
% from big family (4+ paralogs)
60
40
20
0
0
1+
3+
Samples with gene HD
B
*
*
Max. sequence identity
100
75
50
25
0
0
1+
3+
Samples with gene HD
C
OR=0.74*
OR=0.76*
% WGD
80
60
40
20
0
0
1+
3+
Samples with gene HD

Figure 4D

Original Threshold (100)

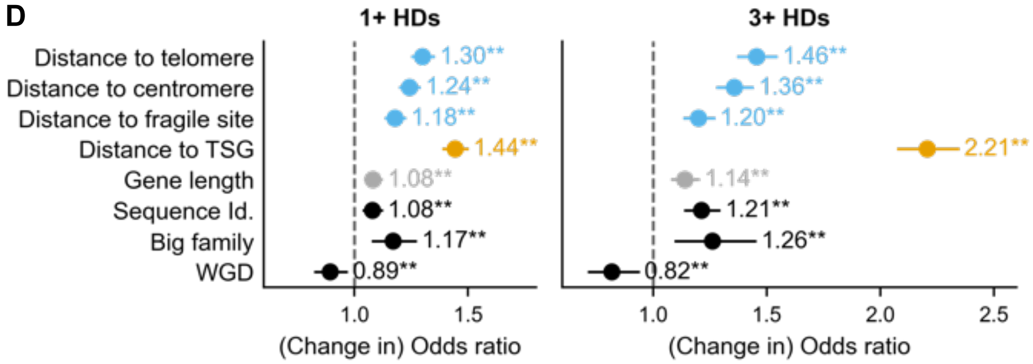

Stricter Threshold (50)

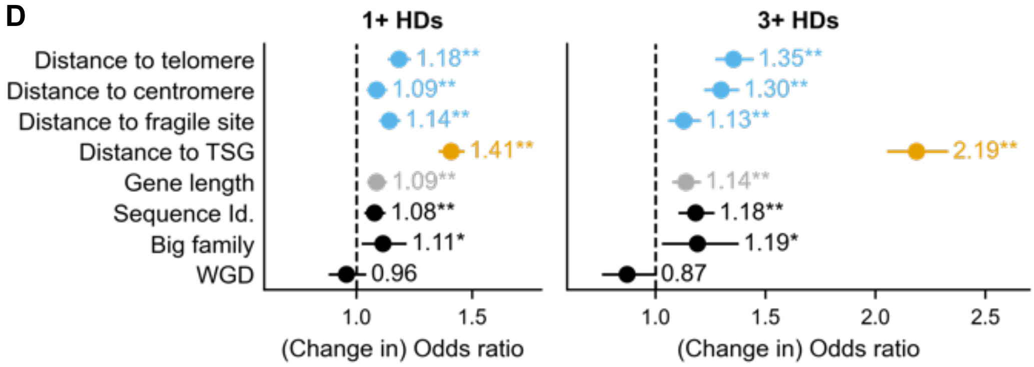

No Threshold

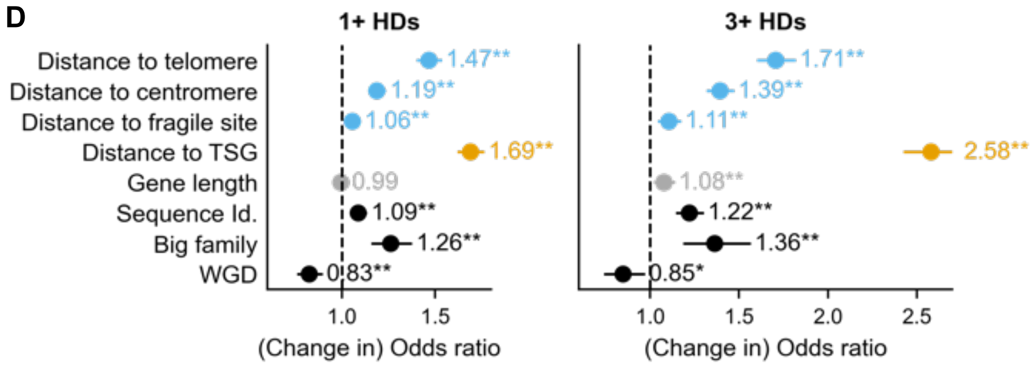

Fig 5 TCGA

Original Threshold (100)

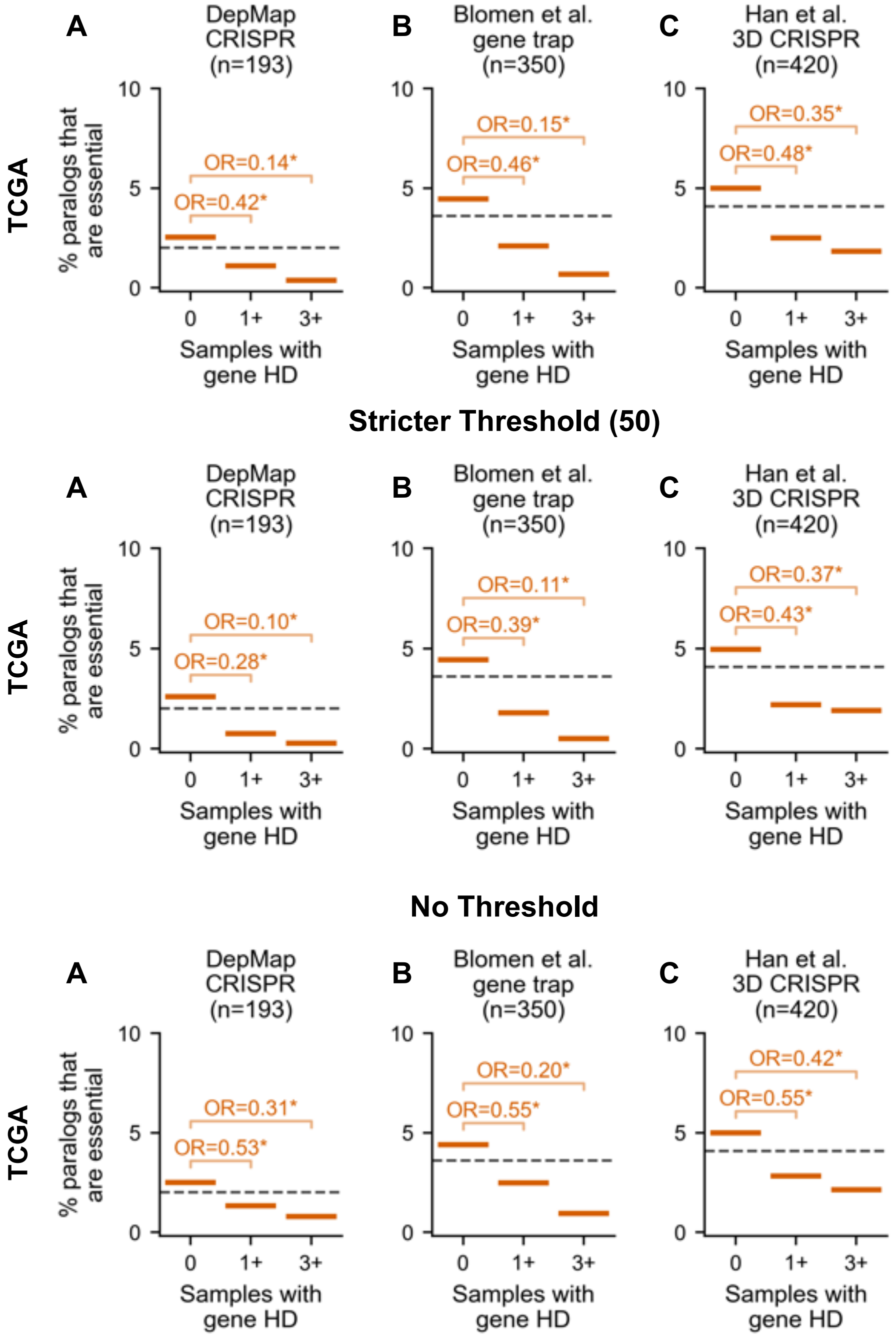

Fig 5 ICGC

Original Threshold (100)

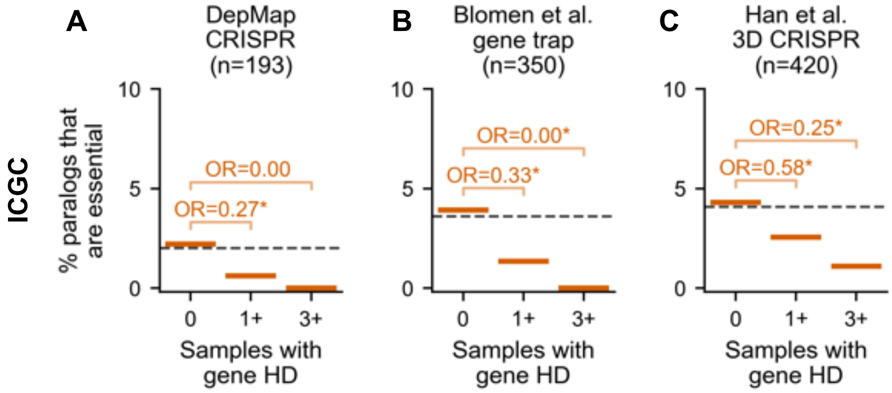

Stricter Threshold (50)

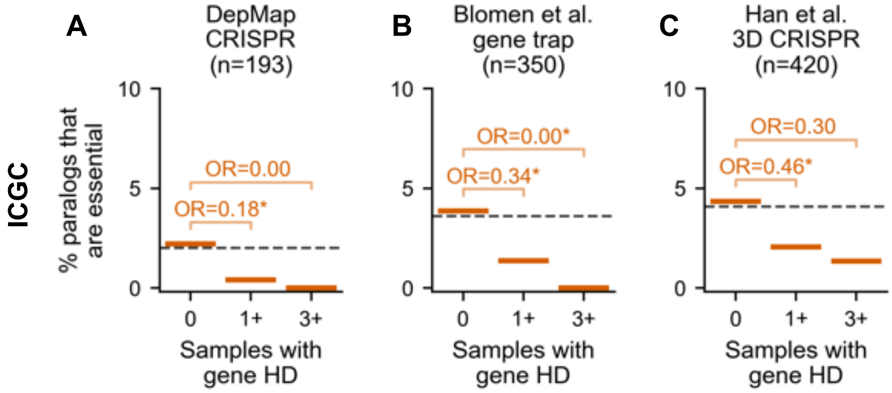

No Threshold

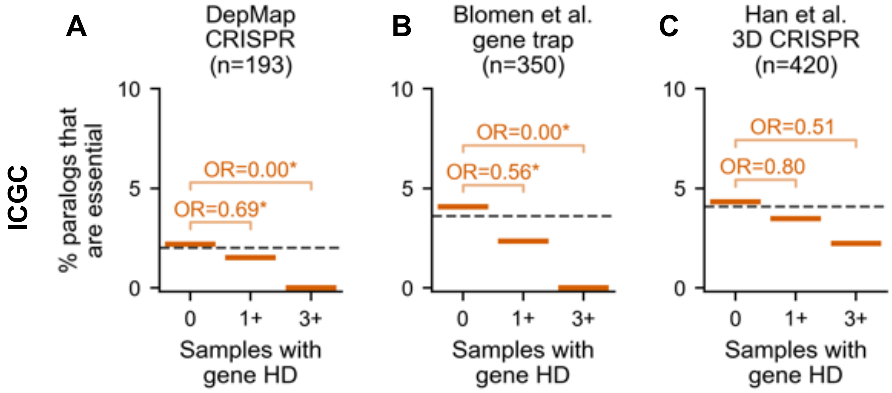

Fig S4

Original Threshold (100)

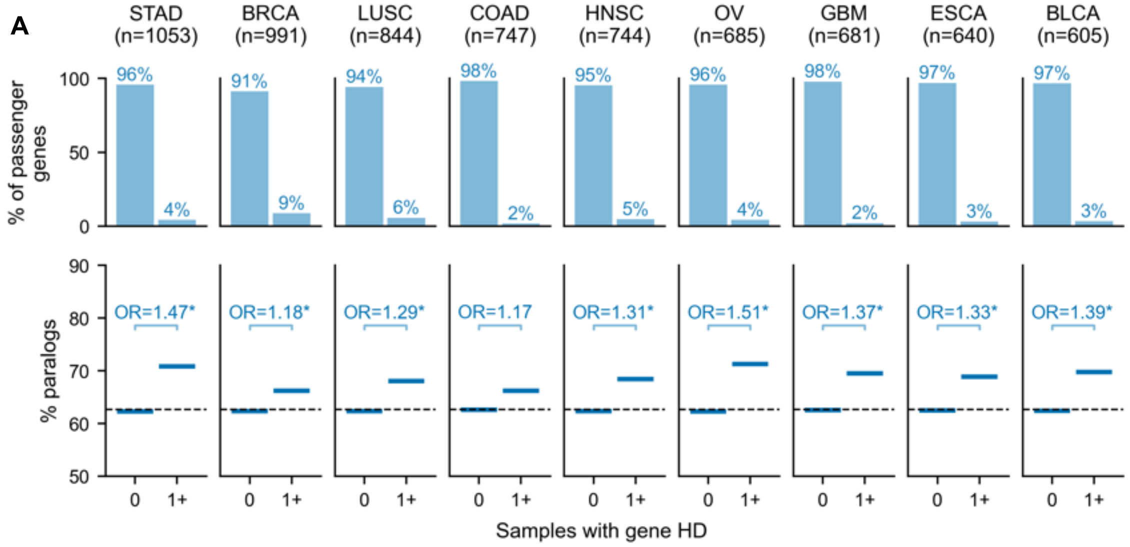

Stricter Threshold (100)

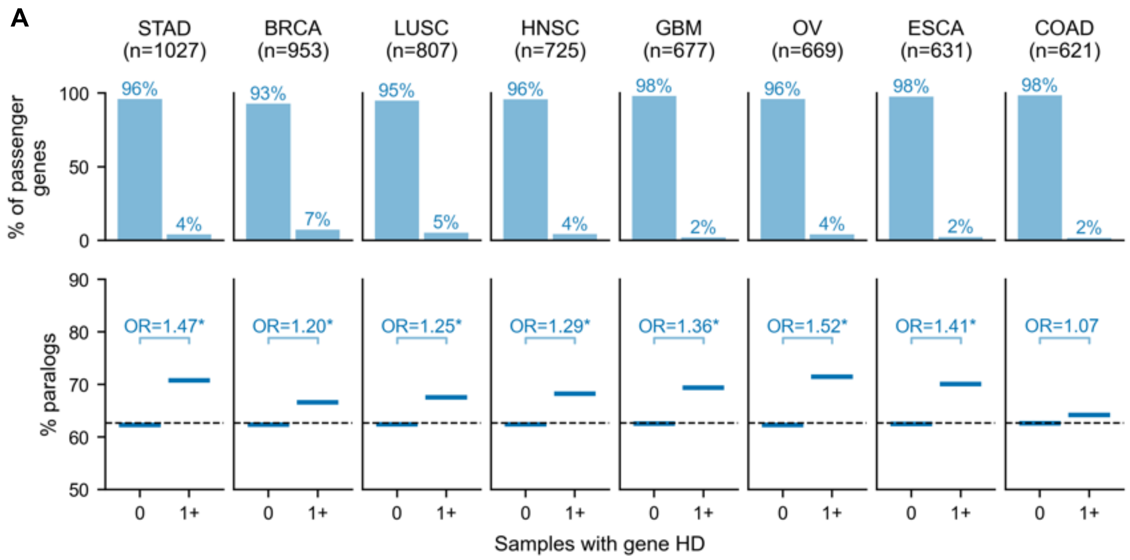

No Threshold

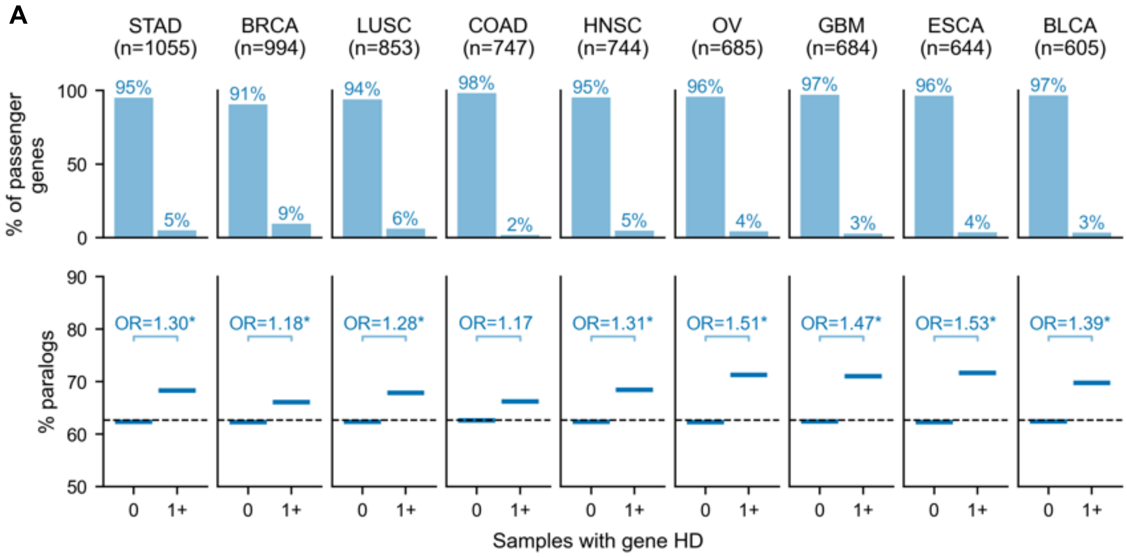

3. Citation for DepMap is missing (caption of Figure 5).

We have added the text below to the legend for Figure 5 :

Essential genes for the DepMap dataset (Meyers et al, 2017) are obtained from a version of the data reprocessed in (De Kegel et al, 2021) to reduce off-target sgRNA effects (see Methods).

CROSS-CONSULTATION COMMENTS
Along the lines of Reviewer #3's second major comment, I have a suggestion, the possible benefits of which would depend on the target audience to which the authors intend to communicate their study.

I would suggest including a brief comparison of the findings of this study which deal with human paralogs, with the findings in model organisms such as yeast, perhaps in the discussion section. To facilitate such a comparison, authors could try measuring the enrichments of, for example, molecular functions, gene families, types of genetic interactions, etc., among the paralogs associated with a high frequency of HDs and then discussing their comparison with what is known in the literature for paralogs in other model organisms that tend to be frequently deleted.

Such a comparison could be of interest to the community of researchers working on other model organisms and put this study in a much broader context. However, as I said before, this would depend on the authors' intended target audience.

We thank the reviewer for the suggestion. We have added an additional section to the discussion highlighting differences and similarities to the observations from yeast as follows:

Much of our understanding of the factors that influence gene dispensability comes from studies in model organisms, in particular the budding yeast Saccharomyces cerevisiae [3,9,10,43,44]. Analyses of the yeast gene deletion collection, a set of gene deletion mutants systematically generated in a single S. cerevisiae strain, revealed that paralogs were less likely to be essential than singleton genes [3,45]. Furthermore, more detailed analyses of yeast paralogs revealed that paralogs from large families were less likely to be essential as were genes with highly sequence similar paralogs [43,44]. Previous analyses, including our own, demonstrated that many of these trends are also evident when analyzing gene essentiality from CRISPR screens in cancer cell lines [12,13,15,35]. Our results here are also consistent with these findings – many of the features that are associated with paralog dispensability in yeast are also associated with gene deletion frequency in tumor genomes.

The connection between the budding yeast observations and those in cancer is less clear when it comes to the relative dispensability of WGDs and SSDs. Analyses of the yeast gene deletion collection revealed that SSDs are more likely to be essential than WGDs in the single genetic background studied [43,44]. In our previous analyses of gene essentiality in hundreds of cancer cell lines we found that SSDs were more likely to be broadly essential (essential in most cell lines) than WGDs but that WGDs were less likely to be never essential (i.e. more likely to be essential in at

least one cell line)[13]. As the analyses of gene essentiality in budding yeast were generated in a single genetic background the concordance with our cancer cell line results was difficult to assess, but as gene deletion collections are now being generated in additional yeast strains it should become possible to perform a more direct comparison[46–48].

Here we found that WGDs are less likely to be deleted than SSDs in tumors. This is surprising in light of the yeast gene deletion collection results, where SSDs were more likely to be essential than WGDs in the strain studied, but less so in light of the cancer cell line results, where WGDs were less likely to be never essential. It is also worth noting that experimental evolution studies in yeast found that SSDs accumulate protein-altering mutations at a higher rate than WGDs [49,50]. These results are perhaps especially relevant when analyzing the influence of paralog features on selection in tumors.

We note that there are many additional differences in the features of WGDs and SSDs in budding yeast that may alter their relative dispensability in tumors. An obvious large scale difference is that in the ancestor of humans there were two rounds of whole genome duplication compared to a single duplication event in yeast[51,52]. Less obvious, but potentially of importance for cancer, is that the two classes of paralogs are enriched in pathways in humans that do not have obvious counterparts in yeast. For example, WGDs are highly enriched in signaling pathways involved in development while SSDs are enriched in immune response genes[53]. How the membership of these pathways influences the dispensability and selection of genes in tumors and cancer cell lines warrants further study.

Reviewer #1 (Significance (Required)):

As the authors note in their manuscript, it is expected that paralog dispensability could be associated with the relaxed negative selection in tumor genomes because (1) paralogs are prevalent in the human genome, and (2) many of them are dispensable, as apparent from the large-scale gene inactivation screens in hundreds of cancer cell lines (Blomen et al. 2015, Wang et al. 2015, Dandage and Landry 2019, De Kegel and Ryan 2019). However, direct mapping of this association, while importantly accounting for potential confounding factors, has been lacking.
As a researcher with prior experience in the research topics such as gene duplication and genetic interactions, it appears to me that this study presents formal proof of the important association between paralog dispensability and tumor genome evolution which could be of major implication for the research community of cancer biology field and specifically to the researchers dealing with the topics such as cancer evolution, copy number alterations in cancer genomes, and synthetic lethality-based precision oncology therapeutics.

Thank you again for the positive assessment.

Reviewer #2 (Evidence, reproducibility and clarity (Required)):

Summary

Here, De Kegel & Ryan analyse thousands of tumour samples from the TCGA and ICGC projects to identify homozygously deleted genes, finding that about 40% of protein-coding genes are deleted in at least one sample. They find homozygously deleted genes to be enriched for paralogous genes, and further, more frequently deleted genes are increasingly likely to be paralogs. The authors then test the influence of several factors on the likelihood of being deleted, including gene length, distance to a fragile site or chromosomal region, and distance to a recurrently deleted tumour suppressor gene (TSG). They find that proximity of a TSG, telomere, centromere, and fragile site all increase likelihood of being deleted in a sample, as does gene length. Having a paralog also remains an important predictor of deletion after accounting for these other factors. Additionally, the more similar in sequence the closest paralog is to the gene and having a larger gene family size are also predictive of deletion. Conversely, if a gene is a whole genome duplicate as opposed to a small-scale duplicate, it is less likely to be deleted. Finally, the authors test the hypothesis that paralogs that are deleted in cancer are less likely to be essential and find that this is indeed the case.

Comments

The authors have done a good job of identifying trends of paralog deletion in cancer samples and the factors influencing them. The results are well described and presented and support the conclusions. I like the inclusion of the saturation analysis as an estimate of what to expect given current and potential future sample sizes, and I appreciate the inclusion of a WGD/SSD paralog distinction. The data and methods are sufficiently detailed. I have a few minor comments below.

We thank the reviewer for the careful reading and positive assessment of our manuscript

1. Around line 160 in the text and supplemental figure 4A, the authors test if the trends they see are observed across individual cancer types. With 9 of 33 cancer types reaching a sample size threshold, 8 of 9 comparisons are significant. The authors do not state correcting for multiple testing.

We have assessed the significance of the results after performing a Holm-Bonferroni correction for multiple hypothesis testing and find that all 8/9 cancer types remain significant.

2. I initially misunderstood the hemizygously deletion analysis, thinking the analysis in supplement figure 4B/C was asking if a sample has any singleton or any paralog deleted and comparing the number of samples with any deletion of either - given the number of genes deleted per sample this wouldn't make sense as a good test. I think the authors are actually comparing the number of loss-of-hemizygosity events per

gene and grouping by paralog/singleton. I think this is a good analysis, but I think it would be helpful to clarify this in the text and figure legend e.g. "Samples w/ gene LOH" could be "LOH events per gene" or something similar.

As suggested we have now updated the y-axis label in these charts to ‘LOH events per gene’. We note that there are now two additional panels in this figure to address copy neutral LOH, per Reviewer 3’s request:

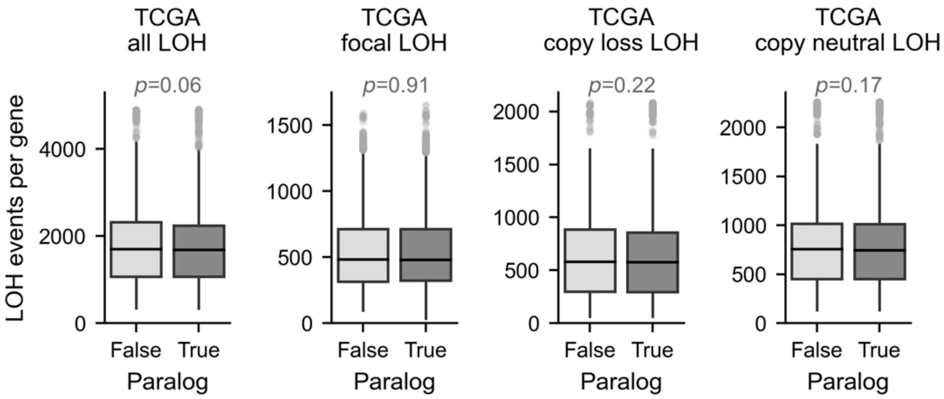

3. Occasionally, I wanted some more detail in the text for context, which was sometimes later provided - e.g. I noted when reading about line 125 that I was curious at this point how often TSGs occurred on segments, and this was later provided on line 241. Similarly, around line 114 I was curious how many genes are typically deleted per HD segment, for which the median value was provided on line 206 (and distribution in supplemental figure 1), and again for hemizygous deletions. I think sometimes it would be helpful to provide this context earlier in the text to aid interpretation of the results.

We thank the reviewer for these suggestions which we have now incorporated into the text.

On line 115 (previously 114) the relevant sentence now reads:

Typically an HD that results in the loss of a protein coding gene also results in the loss of several chromosomally adjacent genes – in the TCGA dataset a median of three genes are lost per gene-deleting HD segment

On line 124 the relevant sentence now reads:

We found that almost half (49%) of the HDs that result in the loss of at least one protein coding gene overlap a known tumor suppressor.

4. In the discussion, on line 420, the authors include the point that a paralog might not be required at all in a tumour cell and therefore easily deleted. I think this possibility could be expanded on here and in the introduction/results section, as it is an important point. I think it would be helpful to include more about the possibility that a paralog might be deleted in a tumour cell because it is simply not required or that is more likely to have less of a phenotypic impact compared to a singleton, and that this could be a reason for the observed enrichment of paralogs in deleted genes.

A citation to support this point could be Áine N O'Toole, Laurence D Hurst, Aoife McLysaght, Faster Evolving Primate Genes Are More Likely to Duplicate, Molecular Biology and Evolution, Volume 35, Issue 1, January 2018, Pages 107-118, https://doi.org/10.1093/molbev/msx270. Duplicate genes can be duplicates because copy number variation of them has minimal impact.

We thank the reviewer for raising this important point.

We have briefly addressed this in the introduction as follows:

In multiple model organisms, paralogs have been demonstrated to be more dispensable than singletons (genes without a paralog) [3–5]. ***There are a number of reasons for why a paralog might be more dispensable than a singleton gene, including preferential retention of duplications of non-essential genes*** *[6,7], but perhaps the most obvious explanation is buffering between paralogs.*

Where references 6 and 7 are as follows:

6. O'Toole ÁN, Hurst LD, McLysaght A. Faster Evolving Primate Genes Are More Likely to Duplicate. Mol Biol Evol. 2018;35: 107–118.
7. He X, Zhang J. Higher duplicability of less important genes in yeast genomes. Mol Biol Evol. 2006;23: 144–151.

We discuss this more comprehensively in the discussion as follows:

In both yeast and cancer there are a number of reasons for why paralogs might be more dispensable than singleton genes. Perhaps the most obvious is the existence of buffering relationships between paralog pairs, such that when one paralog is lost the other paralog can compensate for this loss. Such buffering relationships between paralogs can be revealed through synthetic lethality screens and a number of recurrently deleted paralogs in cancer have already been reported to display synthetic lethal interactions with their paralog (recently reviewed in [54]). Supporting this model, in previous work analysing essentiality in cancer cell lines we found that buffering relationships between paralogs could explain 13-17% of cases where a paralog was essential in some cell lines but not others[13]. This suggests that at least some of the increased dispensability of paralogs in cancer cells can be attributed to buffering relationships between paralog pairs. However this is not the only explanation for paralogs displaying increased dispensability in tumour cells. An additional explanation is that paralogs may perform essential functions in specific contexts (e.g. within specific tissues or at specific developmental stages) but are not required within the specific context of a tumour. Consistent with this model, human paralogs are more likely to display tissue-specific expression patterns [55]. Finally we note that there is evidence to suggest that genes whose perturbation has a lower phenotypic impact may more 'duplicable' – i.e. rather than paralogs being under weaker selection because they are duplicated, their duplication was tolerated because they were already under weaker selection[6,7]. Teasing apart the relative contributions of these factors to the increased dispensability of paralogs in cancer

will require further research and potentially new data resources such as gene essentiality profiles in diverse non-cancer cell types [56].

CROSS-CONSULTATION COMMENTS
I agree, that's a helpful suggestion from reviewer 1.

Reviewer 3's suggestion regarding age of the two whole genome duplication events is quite difficult to unpick as the duplication events seem to have happened relatively close in time to each other while rediploidisation of the first was occurring. Additionally, paralogs from SSDs tend to be more similar in sequence simply because the two WGD events are relatively old while SSDs can occur at any time up to present. They're therefore biased by young duplicates that have not had the opportunity to diverged much and decrease in sequence similarity.

We appreciate these comments.

Reviewer #2 (Significance (Required)):

This is a novel study as it examines the frequency of paralog deletion in cancer samples and the factors influencing it, building upon work already conducted in cancer cell lines. This study extends the knowledge of the field confirming previous trends observed in cell lines, this time in actual cancer samples. It confirms that paralogs are more dispensable than singletons, likely because they have a similar counterpart that can provide some level of functional redundancy. The more similar the closest paralog, the more likely it is to be deleted provides support for this.
It is certainly limited by the number of samples currently available in the two cancer sample projects included but the authors attempt to quantify how limiting this sample size is by conducting a saturation analysis using down-sampling to estimate how many gene deletions one can expect from different numbers of samples. This is important as the lack of observance of many gene deletions is likely due to the limited sample size and not due to negative selection. This low observance of gene deletions disappointingly limits further testing beyond single paralogs to consider the deletion effects of multiple gene family members and more directly test evidence of functional redundancy between paralogs. The authors provide a good discussion of the limitations of their study.

The results are of interest to evolutionary biologists and cancer biologists. Those with an interest in duplicate genes, and/or factors affecting gene loss in tumours will be interested in this work.

My field of expertise is molecular evolution, gene duplication and copy number variation.

We thank the reviewer for the positive assessment of the significance of our work.

Reviewer #3 (Evidence, reproducibility and clarity (Required)):

Thank you review "Paralog dispensability shapes homozygous deletion patterns in tumor genomes" by DeKegel et al. This manuscript uses TCGA and ICGC tumor data to show evidence for paralog dispensability. They analyze the rate of homozygous deletions and show that it is higher for paralogs compared to singletons. Their findings are robust to a number of confounding variables that they take into account e.g. distance to tumor suppressor, telomere, centromere or fragile site. They show that paralogs that belong to large families and have higher sequence identity tend to show more dispensability and these dispensable paralogs are less likely to be WGD.

We thank the reviewer for the time taken to review our manuscript.

Major comments.
1. Does the finding pertaining to lack of enrichment of paralogs in regions LOH take into account whether LOH is copy neutral or not i.e. how does dosage affects this finding? Is it possible that there is a difference in paralog rate in LOH that results in total copy 1 and that the presence of copy neutral LOH masks the effect? Also, Integration of gene expression dataset would be helpful to resolve the difference between dosage paralog that compensate of the lack of their sister by upregulating their gene expression.

In the submitted manuscript we focussed solely on LOH events where the copy number of one allele was 0 and the other allele was ≥1. These include copy loss events (total copy number = 1), copy neutral events (total copy = 2), as well as amplifications (total copy number > 2). The rationale for this approach was that we were interested in understanding whether the mechanism that was generating deletions was preferentially generating deletions in paralog-rich regions.

However, we agree that understanding the influence of dosage is of interest here. We have therefore expanded the analysis in the paper to separately assess the enrichment of paralogs in copy neutral LOH regions (total copy number = 2) and copy loss LOH regions (total copy number = 1).

As shown in the below figure (updated Figure S4B) we do not find an enrichment of paralogs in genes subject to either copy neutral LOH or copy loss LOH.

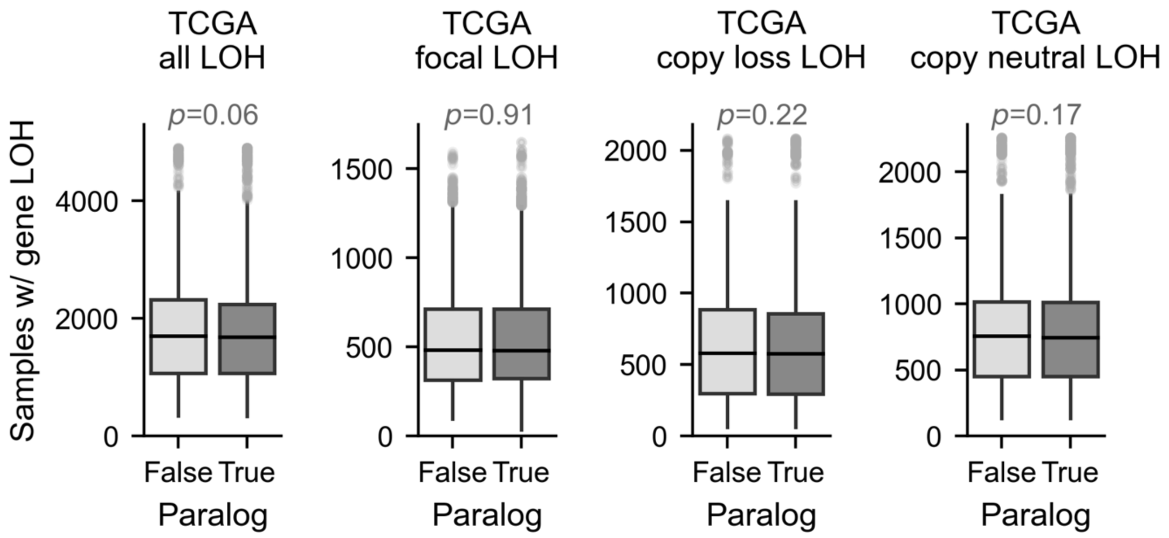

The relevant section of the text on page 6 now reads :

We do not find that paralogs are more frequently subject to LOH than singletons in either the TCGA or ICGC cohort (Fig. S4B-C); when considering all LOH segments we even see that singletons are slightly more frequently subject to LOH in the ICGC cohort (Fig. S4C, left), but when considering only focal LOH segments – i.e. segments whose length is less than half of the chromosome arm's length, which is the case for all HD segments – there is no significant difference between paralog and singleton LOH frequency in either cohort. ***To assess whether gene dosage influenced the observed LOH frequency we further restricted our analysis to copy neutral LOH events (total copy number = 2) and copy loss LOH events (total copy number = 1) and again found no significant increase in deletion frequency of paralogs compared to singletons (Fig. S4B-C).***

Regarding the integration of gene expression to identify dosage compensation between paralogs – we agree that this is extremely interesting and this is something we have been actively working on for quite some time. However, it is quite challenging to address properly. Most paralogs are only observed to be homozygously deleted a single time and so statistically identifying how loss of one gene impacts the mRNA abundance of another is challenging. In the minority of cases where a paralog is recurrently deleted, often these deletions occur in samples from different cancer types and so integrating transcriptomic data still presents some technical challenges. Given this complexity, and as the question of dosage compensation is not central to our key observations, we have not integrated transcriptomic data here.

2. Is the finding that paralogs are depleted among WGD is influenced by the age of WGD since there are 2 WGD events? Do SSD tend to be more or less similar by seq than WGD? This should be explored further since this observation is the opposite of what is observed in model organisms such as yeast whereby SSD are less functionally similar than WGD and often show properties similar to singletons than WGD.

As noted by reviewer 2 in the cross commentary, it is extremely challenging to age the duplicates that arose from the WGD due to the close temporal proximity of the two whole genome duplication events. In the dataset of paralogs analysed used here, SSDs have lower average sequence identity than WGDs. However we note that both sequence identity and duplication type are included in our regression analysis (Figure 4D) and both are significantly associated with homozygous deletion frequently.

This should be explored further since this observation is the opposite of what is observed in model organisms such as yeast whereby SSD are less functionally similar than WGD and often show properties similar to singletons than WGD.

We do not actually think that our results are in opposition to the findings from model organisms. The bulk of studies on the functional consequences of deletions of SSDs/WGDs in model organisms are derived from analyses of the budding yeast gene deletion collection, which is generated in a single strain and grown in lab conditions. Consequently these studies report on which genes can be lost in a single genetic background when grown in rich media. We think it is not fully clear how these findings will apply in the context of a panel of genetically heterogenous tumours derived from multiple different cell types. We note that there are additional complexities when analysing human genes (tissue types, two rounds of WGD, metazoan specific pathways enriched in either WGDs/SSDs) that make a straightforward comparison with yeast challenging. We also note that although the results of analyses of the yeast gene deletion collection suggest that SSDs are more likely to be essential than WGDs, experimental evolution studies have demonstrated that SSDs are more likely to accumulate protein altering mutations than SSDs (Keane et al, Genome Research 2014; Fares et al, PLoS Genetics 2013). This is not what would expect based on the analyses of the yeast gene deletion collection, but is closer to what we observe for tumour genomes where SSDs are more likely to be homozygously deleted.

We agree that we did not adequately discuss these issues in the previous version of our manuscript and so have added a new section to the discussion where we compare our results here with those from budding yeast:

Much of our understanding of the factors that influence gene dispensability comes from studies in model organisms, in particular the budding yeast Saccharomyces cerevisiae [3,9,10,43,44]. Analyses of the yeast gene deletion collection, a set of gene deletion mutants systematically generated in a single S. cerevisiae strain, revealed that paralogs were less likely to be essential than singleton genes [3,45]. Furthermore, more detailed analyses of yeast paralogs revealed that paralogs from large families were less likely to be essential as were genes with highly sequence similar paralogs [43,44]. Previous analyses, including our own, demonstrated that many of these trends are also evident when analyzing gene essentiality from CRISPR screens in cancer cell lines [12,13,15,35]. Our results here are also consistent with these findings – many of the features that are associated with paralog dispensability in yeast are also associated with gene deletion frequency in tumor genomes.

The connection between the budding yeast observations and those in cancer is less clear when it comes to the relative dispensability of WGDs and SSDs. Analyses of the yeast gene deletion collection revealed that SSDs are more likely to be essential than WGDs in the single genetic background studied [43,44]. In our previous analyses of gene essentiality in hundreds of cancer cell lines we found that SSDs were more likely to be broadly essential (essential in most cell lines) than WGDs but that WGDs were less likely to be never essential (i.e. more likely to be essential in at least one cell line)[13]. As the analyses of gene essentiality in budding yeast were generated in a single genetic background the concordance with our cancer cell line results was difficult to assess, but as gene deletion collections are now being generated in additional yeast strains it should become possible to perform a more direct comparison[46–48].

Here we found that WGDs are less likely to be deleted than SSDs in tumors. This is surprising in light of the yeast gene deletion collection results, where SSDs were more likely to be essential than WGDs in the strain studied, but less so in light of the cancer cell line results, where WGDs were less likely to be never essential. It is also worth noting that experimental evolution studies in yeast found that SSDs accumulate protein-altering mutations at a higher rate than WGDs [49,50]. These results are perhaps especially relevant when analyzing the influence of paralog features on selection in tumors.

We note that there are many additional differences in the features of WGDs and SSDs in budding yeast that may alter their relative dispensability in tumors. An obvious large scale difference is that in the ancestor of humans there were two rounds of whole genome duplication compared to a single duplication event in yeast[51,52]. Less obvious, but potentially of importance for cancer, is that the two classes of paralogs are enriched in pathways in humans that do not have obvious counterparts in yeast. For example, WGDs are highly enriched in signaling pathways involved in development while SSDs are enriched in immune response genes[53]. How the membership of these pathways influences the dispensability and selection of genes in tumors and cancer cell lines warrants further study.

Minor comments
1. There is a missing reference on line 55.

We thank the reviewer for catching this oversight. We have now added a reference to Zerbino et al, NAR 2018 on this line.

CROSS-CONSULTATION COMMENTS
That's a good suggestion by reviewer 1. Homozygous deletion collection is available in yeast so these data can be used directly in addition tot he haploid gene deletion collection data.

Since authors of this manuscript included in their analysis the comparison of WGD and SSD then they should do it more thoroughly. It is not sufficient what they presented here especially given that it contradicts the findings from model organisms.

As noted above we have now added a significant discussion of the yeast findings and also of the SSD/WGD observations

Reviewer #3 (Significance (Required)):

This work provides the first systematic assessment of paralog dispensability specifically looking at homozygous deletions of paralogs across primary tumor samples and builds on the existing findings in cancer cell lines. It will be broadly interesting to those studying duplicated gene evolution and genome robustness. My expertise is in complex genetic networks in yeast and human cancer as well as genome evolution.

We thank the reviewer for the positive assessment of our manuscript.

16th Oct 2023

Manuscript Number: MSB-2023-11987
Title: Paralog dispensability shapes homozygous deletion patterns in tumor genomes

Dear Dr. Ryan,

Thank you for submitting your manuscript to Molecular Systems Biology, revised after being reviewed at Review Commons. We have now heard back from the two reviewers who agreed to evaluate your revision. We contacted the same reviewers who evaluated the initial version of the manuscript at Review Commons. Reviewer #1 was not available this time. Reviewer #1 is Review Commons reviewer #3 and reviewer #2 is the same reviewer #2 from Review Commons. As you will see below, both reviewers think that the study has improved as a result of the performed revisions and they support publication. Before we can formally accept the manuscript for publication, we would ask you to address some editorial issues listed below.

- Please provide a .doc version of the manuscript text (including legends for main figures and EV figures) and individual production quality figure files for the main and EV Figures (one file per figure).

- We have replaced Supplementary Information by the Expanded View (EV format). In this case, all additional figures can be provided as EV Figures. Please provide one file per EV Figure. Their legends should be included in the manuscript text. For detailed instructions regarding expanded view please refer to our Author Guidelines: .

- Tables S1-S3 should be provided and called out in the text as Datasets EV1-EV3. Please provide one file per EV Dataset. Each Please include the description of each EV Dataset in the dataset file itself, i.e. in a separate tab for .xls files or as a README.txt file in .zip folders for .csv files.

- Please provide 5 keywords.

- Please provide a "standfirst text" summarizing the study in one or two sentences (approximately 250 characters), three to four "bullet points" highlighting the main findings and a "synopsis image" (550px width and max 400px height, jpeg format) to highlight the paper on our homepage.

- All Materials and Methods need to be described in the main text. We would encourage you to use 'Structured Methods', our new Materials and Methods format. According to this format, the Material and Methods section should include a Reagents and Tools Table (listing key reagents, experimental models, software and relevant equipment and including their sources and relevant identifiers) followed by a Methods and Protocols section in which we encourage the authors to describe their methods using a step-by-step protocol format with bullet points, to facilitate the adoption of the methodologies across labs. More information on how to adhere to this format as well as downloadable templates (.doc or .xls) for the Reagents and Tools Table can be found in our author guidelines: . An example of a Method paper with Structured Methods can be found here:

- Please include a Data availability section, structured according to the example below:
The datasets and computer code produced in this study are available in the following databases:
- Chip-Seq data: Gene Expression Omnibus GSE46748 (https://www.ncbi.nlm.nih.gov/geo/query/acc.cgi?acc=GSE46748)
- Modeling computer scripts: GitHub (https://github.com/SysBioChalmers/GECKO/releases/tag/v1.0)
- [data type]: [full name of the resource] [accession number/identifier] ([doi or URL or identifiers.org/DATABASE:ACCESSION])

- For data quantification: please specify the name of the statistical test used to generate error bars and P values, the number (n) of independent experiments (specify technical or biological replicates) underlying each data point and the test used to calculate p-values in each figure legend. The figure legends should contain a basic description of n, P and the test applied. Graphs must include a description of the bars and the error bars (s.d., s.e.m.).

- The References should be formatted according to the Molecular Systems Biology reference style (i.e. ordered alphabetically and listing the first 10 authors followed by et al).

- Please include a "Disclosure and Competing Interests statement" in the main text.

- Molecular Systems Biology supports formal data citations in the Reference list, to cite previously published datasets. In addition to citing the original papers that reported the data, we encourage you to also cite the relevant datasets directly in the Reference list. In the text, references to datasets are included as "Data ref: Smith et al, 2001" or "Data ref: NCBI Sequence Read Archive PRJNA342805, 2017". In the Reference list, data citations are very similar to normal literature references but must be labeled with "[DATASET]" at the end of the reference. For detailed instructions please refer to our Author Guidelines .

- When you resubmit your manuscript, please download our CHECKLIST (https://bit.ly/EMBOPressAuthorChecklist) and include

the completed form in your submission.
Please note that the Author Checklist will be published alongside the paper as part of the transparent process (https://www.embopress.org/page/journal/17444292/authorguide#transparentprocess).

Please resubmit your revised manuscript online, with a covering letter listing amendments and responses to each point raised by the referees. Please resubmit the paper **within one month** and ideally as soon as possible. If we do not receive the revised manuscript within this time period, the file might be closed and any subsequent resubmission would be treated as a new manuscript. Please use the Manuscript Number (above) in all correspondence.

Click on the link below to submit your revised paper.

Link Not Available

As a matter of course, please make sure that you have correctly followed the instructions for authors as given on the submission website.

Thank you for submitting this paper to Molecular Systems Biology.

Kind regards,

Maria

Maria Polychronidou, PhD
Senior Editor
Molecular Systems Biology

If you do choose to resubmit, please click on the link below to submit the revision online before 15th Nov 2023.

Link Not Available

IMPORTANT: When you send your revision, we will require the following items:
1. the manuscript text in LaTeX, RTF or MS Word format
2. a letter with a detailed description of the changes made in response to the referees. Please specify clearly the exact places in the text (pages and paragraphs) where each change has been made in response to each specific comment given
3. three to four 'bullet points' highlighting the main findings of your study
4. a 'standfirst text' summarizing in two sentences the study (approx. 250 characters)
6. a "thumbnail image" (width=211 x height=157 pixels, jpeg format), which can be used as 'visual title' to highlight your paper on our homepage.
7. Please include an author contributions statement after the Acknowledgements section (see https://www.nature.com/msb/authors/index.html#Submission)
8. When assembling figures, please refer to our figure preparation guideline in order to ensure proper formatting and readability in print as well as on screen:
https://bit.ly/EMBOPressFigurePreparationGuideline
See also figure legend guidelines: https://www.embopress.org/page/journal/17444292/authorguide#figureformat

*** PLEASE NOTE *** As part of the EMBO Publications transparent editorial process initiative (see our Editorial at https://www.nature.com/msb/journal/v6/n1/full/msb201072.html), Molecular Systems Biology will publish online a Review Process File to accompany accepted manuscripts. When preparing your letter of response, please be aware that in the event of acceptance, your cover letter/point-by-point document will be included as part of this File, which will be available to the scientific community. More information about this initiative is available in our Instructions to Authors. If you have any questions about this initiative, please contact the editorial office (msb@embo.org).

Reviewer #1:

Authors sufficiently addressed most comments. This manuscript should be accepted.

Reviewer #2:

Thank you for implementing the suggested changes from our previous review round. This reviewer is now satisfied with the

comprehensive responses from the authors and amendments that have made to their article following our comments.
I suggest it is now fit and well suited for publication in Molecular Systems Biology.

***
Rev_Com_number: RC-2022-01743
New_manu_number: MSB-2023-11987
Corr_author: Ryan
Title: Paralog dispensability shapes homozygous deletion patterns in tumor genomes

Reviewer #1:

Authors sufficiently addressed most comments. This manuscript should be accepted.

We thank the reviewer for the positive response and for all of the feedback on our manuscript.

Reviewer #2:

Thank you for implementing the suggested changes from our previous review round.
This reviewer is now satisfied with the comprehensive responses from the authors and amendments that have made to their article following our comments.
I suggest it is now fit and well suited for publication in Molecular Systems Biology.

We thank the reviewer for the positive response and for all of the feedback on our manuscript.

26th Oct 2023

Manuscript number: MSB-2023-11987R
Title: Paralog dispensability shapes homozygous deletion patterns in tumor genomes

Dear Dr Ryan,

Thank you again for sending us your revised manuscript. We are now satisfied with the modifications made and I am pleased to inform you that your paper has been accepted for publication.

*** PLEASE NOTE *** As part of the EMBO Publications transparent editorial process initiative (see our Editorial at https://dx.doi.org/10.1038/msb.2010.72), Molecular Systems Biology publishes online a Review Process File with each accepted manuscripts. This file will be published in conjunction with your paper and will include the anonymous referee reports, your point- by-point response and all pertinent correspondence relating to the manuscript. If you do NOT want this File to be published, please inform the editorial office at msb@embo.org within 14 days upon receipt of the present letter.

Should you be planning a Press Release on your article, please get in contact with msb@wiley.com as early as possible, in order to coordinate publication and release dates.

LICENSE AND PAYMENT:
All articles published in Molecular Systems Biology are fully open access: immediately and freely available to read, download and share.

Molecular Systems Biology charges an article processing charge (APC) to cover the publication costs. You, as the corresponding author for this manuscript, should have already received a quote with the article processing fee separately. Please let us know in case this quote has not been received.

Once your article is at Wiley for editorial production you will receive an email from Wiley's Author Services system, which will ask you to log in and will present you with the publication license form for completion. Within the same system the publication fee can be paid by credit card, an invoice or pro forma can be requested.

Payment of the publication charge and the signed Open Access Agreement form must be received before the article can be published online.

Molecular Systems Biology articles are published under the Creative Commons licence CC BY, which facilitates the sharing of scientific information by reducing legal barriers, while mandating attribution of the source in accordance to standard scholarly practice.

Proofs will be forwarded to you within the next 2-3 weeks.

Thank you very much for submitting your work to Molecular Systems Biology.

Kind regards,

Maria

Maria Polychronidou, PhD
Senior Editor
Molecular Systems Biology

Click here to be directed to your login page: https://msb.msubmit.net

***
Rev_Com_number: RC-2022-01743
New_manu_number: MSB-2023-11987R
Corr_author: Ryan
Title: Paralog dispensability shapes homozygous deletion patterns in tumor genomes

EMBO Press Author Checklist

Corresponding Author Name: Colm Ryan
Journal Submitted to: Molecular Systems Biology
Manuscript Number: MSB-2023-11987

USEFUL LINKS FOR COMPLETING THIS FORM
The EMBO Journal - Author Guidelines
EMBO Reports - Author Guidelines
Molecular Systems Biology - Author Guidelines
EMBO Molecular Medicine - Author Guidelines

Reporting Checklist for Life Science Articles (updated January
This checklist is adapted from Materials Design Analysis Reporting (MDAR) Checklist for Authors. MDAR establishes a minimum set of requirements in transparent reporting in the life sciences (see Statement of Task: 10.31222/osf.io/9sm4x). Please follow the journal's guidelines in preparing your
Please note that a copy of this checklist will be published alongside your article.

Abridged guidelines for figures
1. Data
The data shown in figures should satisfy the following conditions:
- the data were obtained and processed according to the field's best practice and are presented to reflect the results of the experiments in an accurate and unbiased manner.
- ideally, figure panels should include only measurements that are directly comparable to each other and obtained with the same assay.
- plots include clearly labeled error bars for independent experiments and sample sizes. Unless justified, error bars should not be shown for technical
- if n<5, the individual data points from each experiment should be plotted. Any statistical test employed should be justified.
- Source Data should be included to report the data underlying figures according to the guidelines set out in the authorship guidelines on Data

2. Captions
Each figure caption should contain the following information, for each panel where they are relevant:
- a specification of the experimental system investigated (eg cell line, species name).
- the assay(s) and method(s) used to carry out the reported observations and measurements.
- an explicit mention of the biological and chemical entity(ies) that are being measured.
- an explicit mention of the biological and chemical entity(ies) that are altered/varied/perturbed in a controlled manner.
- the exact sample size (n) for each experimental group/condition, given as a number, not a range;
- a description of the sample collection allowing the reader to understand whether the samples represent technical or biological replicates (including how many animals, litters, cultures, etc.).
- a statement of how many times the experiment shown was independently replicated in the laboratory.
- definitions of statistical methods and measures:
  - common tests, such as t-test (please specify whether paired vs. unpaired), simple χ2 tests, Wilcoxon and Mann-Whitney tests, can be unambiguously identified by name only, but more complex techniques should be described in the methods section;
  - are tests one-sided or two-sided?
  - are there adjustments for multiple comparisons?
  - exact statistical test results, e.g., P values = x but not P values < x;
  - definition of 'center values' as median or average;
  - definition of error bars as s.d. or s.e.m.

Please complete ALL of the questions below.
Select "Not Applicable" only when the requested information is not relevant for your study.

Materials

Newly Created Materials	Information included in the manuscript?	In which section is the information available? (Reagents and Tools Table, Materials and Methods, Figures, Data Availability Section)
New materials and reagents need to be available; do any restrictions apply?	Not Applicable	

Antibodies	Information included in the manuscript?	In which section is the information available? (Reagents and Tools Table, Materials and Methods, Figures, Data Availability Section)
For **antibodies** provide the following information:<br>- Commercial antibodies: RRID (if possible) or supplier name, catalogue number and or/clone number<br>- Non-commercial: RRID or citation	Not Applicable	

DNA and RNA sequences	Information included in the manuscript?	In which section is the information available? (Reagents and Tools Table, Materials and Methods, Figures, Data Availability Section)
**Short novel DNA or RNA including primers, probes**: provide the sequences.	Not Applicable	

Cell materials	Information included in the manuscript?	In which section is the information available? (Reagents and Tools Table, Materials and Methods, Figures, Data Availability Section)
**Cell lines:** Provide species information, strain. Provide accession number in repository **OR** supplier name, catalog number, clone number, and/**OR** RRID.	Not Applicable	
**Primary cultures:** Provide species, strain, sex of origin, genetic modification status.	Not Applicable	
Report if the cell lines were recently **authenticated** (e.g., by STR profiling) and tested for mycoplasma contamination.	Not Applicable	

Experimental animals	Information included in the manuscript?	In which section is the information available? (Reagents and Tools Table, Materials and Methods, Figures, Data Availability Section)
**Laboratory animals or Model organisms:** Provide species, strain, sex, age, genetic modification status. Provide accession number in repository **OR** supplier name, catalog number, clone number, **OR** RRID.	Not Applicable	
**Animal observed in or captured from the field:** Provide species, sex, and age where possible**.**	Not Applicable	
Please detail **housing and husbandry conditions**.	Not Applicable	

Plants and microbes	Information included in the manuscript?	In which section is the information available? (Reagents and Tools Table, Materials and Methods, Figures, Data Availability Section)
**Plants:** provide species and strain, ecotype and cultivar where relevant, unique accession number if available, and source (including location for collected wild specimens).	Not Applicable	
**Microbes:** provide species and strain, unique accession number if available, and source.	Not Applicable	

Human research participants	Information included in the manuscript?	In which section is the information available? (Reagents and Tools Table, Materials and Methods, Figures, Data Availability Section)
If collected and within the bounds of privacy constraints report on age, sex and gender or ethnicity for all study participants.	Not Applicable	

Core facilities	Information included in the manuscript?	In which section is the information available? (Reagents and Tools Table, Materials and Methods, Figures, Data Availability Section)
If your work benefited from core facilities, was their service mentioned in the acknowledgments section?	Not Applicable	

Design

Study protocol	Information included in the manuscript?	In which section is the information available? (Reagents and Tools Table, Materials and Methods, Figures, Data Availability Section)
If study protocol has been **pre-registered, provide DOI in the manuscript**. For clinical trials, provide the trial registration number **OR** cite DOI.	Not Applicable	
Report the **clinical trial registration number** (at ClinicalTrials.gov or equivalent), where applicable.	Not Applicable	

Laboratory protocol	Information included in the manuscript?	In which section is the information available? (Reagents and Tools Table, Materials and Methods, Figures, Data Availability Section)
Provide DOI OR other citation details if **external detailed step-by-step protocols** are available.	Not Applicable	

Experimental study design and statistics	Information included in the manuscript?	In which section is the information available? (Reagents and Tools Table, Materials and Methods, Figures, Data Availability Section)
Include a statement about **sample size** estimate even if no statistical methods were used.	Not Applicable	
Were any steps taken to minimize the effects of subjective bias when allocating animals/samples to treatment (e.g. **randomization procedure**)? If yes, have they been described?	Not Applicable	
Include a statement about **blinding** even if no blinding was done.	Not Applicable	
Describe **inclusion/exclusion criteria** if samples or animals were excluded from the analysis. Were the criteria pre-established?<br><br>If sample or data points were omitted from analysis, report if this was due to attrition or intentional exclusion and provide justification.	Yes	Tumour samples with extreme numbers of deletions were excluded, discussed in Materials and Methods and presented in Fig EV1
For every figure, are **statistical tests** justified as appropriate? Do the data meet the assumptions of the tests (e.g., normal distribution)? Describe any methods used to assess it. Is there an estimate of variation within each group of data? Is the variance similar between the groups that are being statistically compared?	Yes	Materials and Methods, Figure Legends

Sample definition and in-laboratory replication	Information included in the manuscript?	In which section is the information available? (Reagents and Tools Table, Materials and Methods, Figures, Data Availability Section)
In the figure legends: state number of times the experiment was **replicated** in laboratory.	Not Applicable	
In the figure legends: define whether data describe **technical or biological replicates**.	Not Applicable	

Ethics

Ethics	Information included in the manuscript?	In which section is the information available? (Reagents and Tools Table, Materials and Methods, Figures, Data Availability Section)
Studies involving **human participants**: State details of **authority granting ethics approval** (IRB or equivalent committee(s), provide reference number for approval.	Not Applicable	
Studies involving **human participants**: Include a statement confirming that **informed consent** was obtained from all subjects and that the experiments conformed to the principles set out in the WMA Declaration of Helsinki and the Department of Health and Human Services Belmont Report.	Not Applicable	
Studies involving **human participants:** For publication of **patient photos**, include a statement confirming that consent to publish was obtained.	Not Applicable	
Studies involving experimental **animals**: State details of **authority granting ethics approval** (IRB or equivalent committee(s), provide reference number for approval. Include a statement of compliance with ethical regulations.	Not Applicable	
Studies involving **specimen and field samples:** State if relevant **permits** obtained, provide details of authority approving study; if none were required, explain why.	Not Applicable	

Dual Use Research of Concern (DURC)	Information included in the manuscript?	In which section is the information available? (Reagents and Tools Table, Materials and Methods, Figures, Data Availability Section)
Could your study fall under dual use research restrictions? Please check biosecurity documents and list of **select agents and toxins** (CDC): https://www.selectagents.gov/sat/list.htm	Not Applicable	
If you used a select agent, is the security level of the lab appropriate and reported in the manuscript?	Not Applicable	
If a study is subject to dual use research of concern regulations, is the name of the **authority granting approval and reference number** for the regulatory approval provided in the manuscript?	Not Applicable	

Reporting

The MDAR framework recommends adoption of discipline-specific guidelines, established and endorsed through community initiatives. Journals have their own policy about requiring specific guidelines and recommendations to complement MDAR.

Adherence to community standards	Information included in the manuscript?	In which section is the information available? (Reagents and Tools Table, Materials and Methods, Figures, Data Availability Section)
State if relevant guidelines or checklists (e.g., **ICMJE, MIBBI, ARRIVE, PRISMA**) have been followed or provided.	Not Applicable	
For **tumor marker prognostic studies,** we recommend that you follow the **REMARK** reporting guidelines (see link list at top right). See author guidelines, under 'Reporting Guidelines'. Please confirm you have followed these guidelines.	Not Applicable	
For **phase II and III randomized controlled trials**, please refer to the **CONSORT** flow diagram (see link list at top right) and submit the CONSORT checklist (see link list at top right) with your submission. See author guidelines, under 'Reporting Guidelines'. Please confirm you have submitted this list.	Not Applicable	

Data Availability

Data availability	Information included in the manuscript?	In which section is the information available? (Reagents and Tools Table, Materials and Methods, Figures, Data Availability Section)
Have **primary datasets** been deposited according to the journal's guidelines (see 'Data Deposition' section) and the respective accession numbers provided in the Data Availability Section?	Not Applicable	
Were **human clinical and genomic datasets** deposited in a public access-controlled repository in accordance to ethical obligations to the patients and to the applicable consent agreement?	Not Applicable	
Are **computational models** that are central and integral to a study available without restrictions in a machine-readable form? Were the relevant accession numbers or links provided?	Not Applicable	
If publicly available data were reused, provide the respective **data citations in the reference list**.	Yes	Materials and Methods
